# Analysis of research trends and development prospects of soluble guanylate cyclase stimulators/activators: using bibliometric methods

**DOI:** 10.3389/fphar.2025.1501330

**Published:** 2025-06-10

**Authors:** Bing Ju Yan, Jing Zhang, Jun Wang, Hong Xia Yuan

**Affiliations:** ^1^ Department of Cardiology, The First Affiliated Hospital of Jinzhou Medical University, Jinzhou, China; ^2^ Department of General Surgery, The First Affiliated Hospital of Jinzhou Medical University, Jinzhou, China; ^3^ Division of Infectious Diseases, The First Affiliated Hospital of Jinzhou Medical University, Jinzhou, China

**Keywords:** bibliometric, soluble guanylate cyclase (sGC) stimulator, soluble guanylate cyclase (sGC) activator, riociguat, vericiguat

## Abstract

**Background:**

The stimulation and activation of soluble guanylate cyclase (sGC) has been a prominent focus of study in recent years. Currently, sGC stimulators/activators have a wide range of applications in various fields. However, there remains a gap in bibliometric analysis on this topic. We aim to visualize the research hotspots and trends in the field of sGC stimulators or activators. This analysis can equip researchers to understand current findings and forecast future trends efficiently.

**Methods:**

The researchers searched for publications on sGC stimulators or activators in Web of Science Core Collection (WoSCC) on 22 July 2024. We then organized and analyzed the data using Microsoft Excel, online tool (Weishengxin), R, VOS viewer, and CiteSpace.

**Results:**

1,879 papers from WoSCC were gathered for analysis, comprising 1,582 articles and 297 reviews. The United States is the most productive country. And Germany stands out as the country with the most robust collaboration. The most productive institution is BAYER AG. Furthermore, the majority of journals with citation frequencies in the top 10 belong to Q1. The Professor Stasch, JP is the most prolific author and the top co-cited author. Currently, the influential keywords include “riociguat”, “outcome”, “guanylate cyclase stimulator”, “heart failure”, “natriuretic peptide”, “vericiguat”, “reduced ejection fraction”, “mortality” and “placebo”.

**Conclusion:**

The future research hotspots will focus on the following aspects based on the current research hotspots: the safety verification of riociguat, the clinical efficacy of vericiguat for other types of heart failure, the role of praliciguat in diabetes nephropathy, and the efficacy and safety of newly discovered drugs. Furthermore, actively exploring new therapeutic directions for sGC stimulators or activators may also be an important trend in the future development of this field.

## 1 Introduction

Since 1958, bibliometrics has been widely recognized and has developed into a distinct field of analysis (Thelwall, 2008). It is grounded in the theoretical foundations of Lotka’s Law, Zipf’s Law, and Bradford’s Law, employing a variety of analytical methods such as citation analysis, co-word analysis, cluster analysis, time series analysis, and visualization analysis to conduct detailed and comprehensive research on selected literature. This approach presents broad development prospects. Citation analysis is one of the core methods in bibliometric analysis. It can not only count the citation frequency and citation frequency of literature, evaluate the academic influence and value of literature, but also construct citation networks to reveal the relationships and academic inheritance between literature. Co-word analysis mainly reveals the research hotspots and topic structures in a discipline by analyzing the co-occurrence of different keywords in the same document. It can also visually display the association between keywords, helping researchers quickly locate the core concepts and research directions within the field. Cluster analysis is a statistical analysis method that classifies similar objects. By clustering literature, literature with similar topics and research directions can be grouped together, revealing the knowledge structure and research hotspots within the discipline, providing strong support for scientific research topics and literature reviews. Time series analysis is a statistical method to study the trend of data change over time. In bibliometrics analysis, time series analysis is used to reveal the development law and trend of the subject field over time, and it provides an important reference for researchers to formulate discipline development strategy and research planning. Visual analysis is an analysis method that uses visualization software (such as CiteSpace, etc.) to display the analysis results in graphics, images and other intuitive forms, which can help researchers better understand and analyze data. In summary, bibliometrics is a discipline that uses statistical analysis to analyze and visually represent the distribution and variability of quantitative features in literature. Through bibliometric research, scholars can efficiently gain a comprehensive understanding of the research in their respective fields. This facilitates a better grasp of research hotspots, enables them to keep abreast of developing trends, and helps in predicting future research areas and directions (Broadus, 1987; Borgman and Furner, 2002; Donthu et al., 2021).

The soluble guanylate cyclase (sGC) is a vital enzyme in the nitric oxide (NO) signal transduction pathway. It plays a crucial role in activating the NO-sGC-cGMP signaling pathway and also has the capability to inhibit the TGF-β signaling pathway. Activation of the NO-sGC-cGMP pathway can stimulate the production of cGMP in the body. cGMP, an essential second messenger in the body (Friebe et al., 2020), is involved in regulating downstream effecting molecules and participating in various physiological or pathological reactions. Inhibition of the TGF-β pathway can have physiological effects by inhibiting tissue fibrosis and cell proliferation (Xiao et al., 2019). has emerged as a novel therapeutic target for cardiopulmonary diseases (Stasch et al., 2011). Research on sGC stimulators or activators has been ongoing since the 1990s.

YC-1 is an sGC activator discovered in the mid-1990s (Mulsch et al., 1997), which has anti thrombotic effects *in vivo* (Ko et al., 1994; Wu et al., 1995). It mainly induces sGC activation by binding to allosteric sites on sGC molecules, thereby enhancing the sensitivity of sGC to gaseous activators (Friebe and Koesling, 1998). Subsequently, researchers found that YC-1 is not only an activator of sGC, but also appears to be a HIF-1 inhibitor (Yeo et al., 2003; Sun et al., 2007). It can exert anti-cancer effects by inhibiting the activity of HIF-1 and suppressing the expression of vascular endothelial growth factor (Yeo et al., 2004).

In 2013, with the approval of riociguat as the first sGC stimulator, research reached a significant milestone. Riociguat not only significantly improves exercise performance in patients with pulmonary hypertension (PH), regardless of whether the patients have used endothelin receptor antagonists or prostaglandins, but also enhances secondary efficacy endpoints such as pulmonary hemodynamics, WHO functional grade, and clinical deterioration time (Ghofrani et al., 2013b).

The novel drug vericiguat, the first sGC stimulator developed for the treatment of patients with worsening chronic heart failure, received approval from the FDA for market release in January 2021. Upon its release to the market, the drug quickly garnered significant attention from researchers. Vericiguat is a groundbreaking oral soluble guanylate cyclase stimulator capable of directly generating cyclic guanosine monophosphates and restoring the sensitivity of soluble guanylate cyclase to nitric oxide (Markham and Duggan, 2021). Multiple clinical trials have demonstrated its ability to reduce cardiovascular mortality in patients with heart failure with reduced ejection fraction (HFrEF) (Pieske et al., 2017; Armstrong et al., 2020b). It has also been shown to improve the quality of life for heart failure (HF) patients, with excellent tolerability and safety (Zheng et al., 2018; Liang and Liang, 2023).

At present, the sGC stimulators or activators have been shown to possess a range of therapeutic effects, including the treatment of HF and PH (Koress et al., 2016; Zheng et al., 2018; Sandner et al., 2021a; Ruopp and Cockrill, 2022), as well as playing a protective role in kidney function (Stasch et al., 2015). (Especially in recent years, the prevalence of diabetes has increased dramatically worldwide, and its complication, diabetic nephropathy, has become the main culprit of end-stage renal disease (Koye et al., 2018). Therefore, it is crucial to explore the renal protection effects of sGC stimulators or activators). SGC stimulators or activators can also enhance the susceptibility of head squamous cell carcinoma to cisplatin (Tuttle et al., 2017). Additionally, some researchers have proposed the feasibility of sGC stimulators or activators as a new potential method for conjunctiva fibrosis or pulmonary fibrosis through experimental studies based on the effect of sGC inhibiting TGF-β pathway on tissue fibrosis and cell proliferation (Li et al., 2023; Fioretto et al., 2024). Some researchers have also pointed out that sGC stimulators or activators have the effect of enhancing the body’s memory (Nelissen et al., 2021). Although not currently in widespread use, they present a new potential for the advancement of sGC stimulators or activators. Significant progress and achievements have been made in the studies of sGC stimulators or activators up to this point. However, to the best of our knowledge, there remains a significant gap in bibliometric research on sGC stimulators or activators. Therefore, we firmly believe that conducting research in this area is essential in order to provide valuable information and new directions for researchers.

## 2 Data and methods

### 2.1 Data sources and search strategies

**FIGURE 1:**
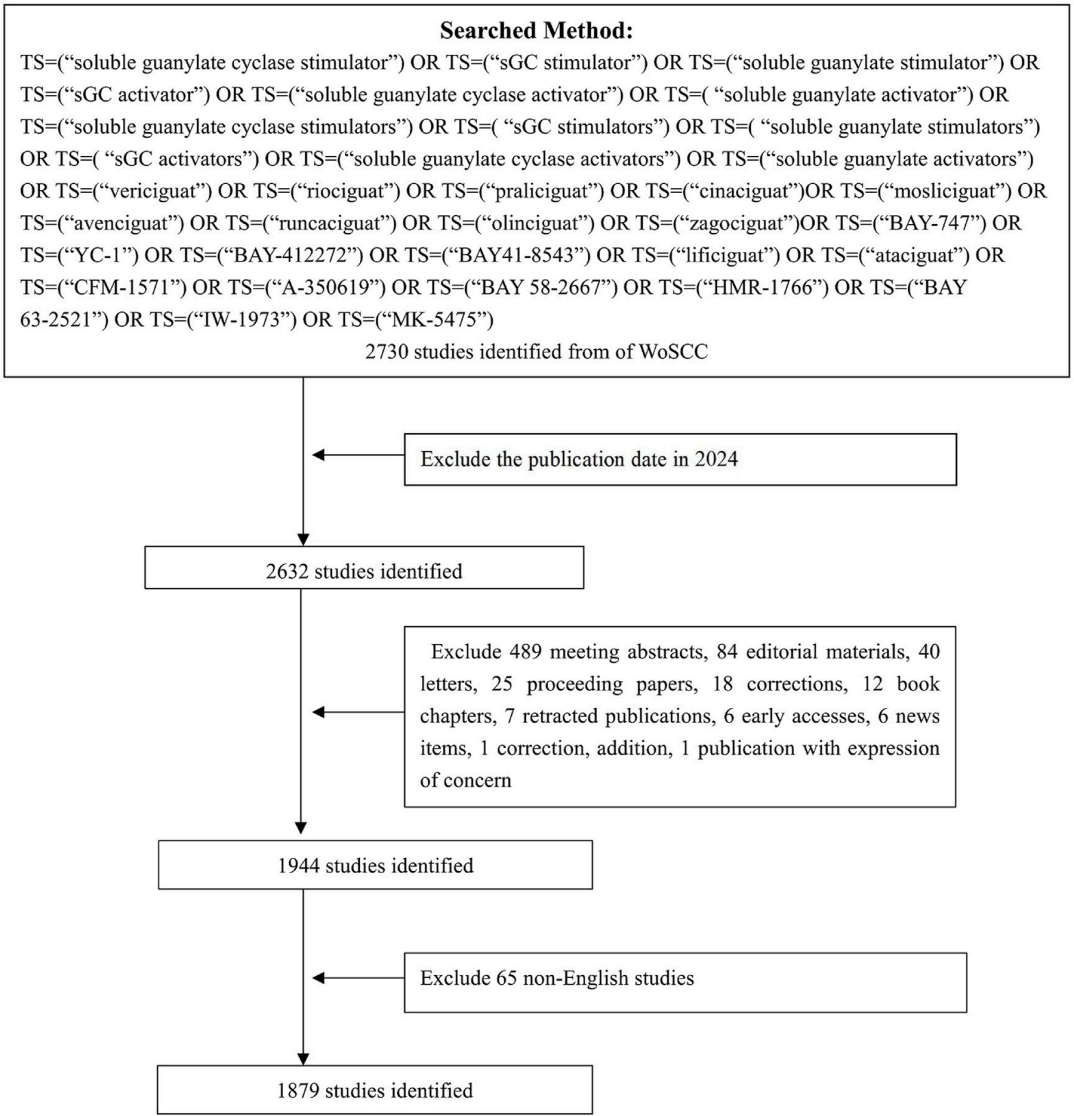
Flowchart of the screening process.

We selected the WoSCC database for our research. The researchers conducted a search and downloaded the data from the database on 22 July 2024. The search strategy was as follows: TS = (“soluble guanylate cyclase stimulator”) OR TS = (“sGC stimulator”) OR TS = (“soluble guanylate stimulator”) OR TS = (“sGC activator”) OR TS = (“soluble guanylate cyclase activator”) OR TS = (“soluble guanylate activator”) OR TS = (“soluble guanylate cyclase stimulators”) OR TS = (“sGC stimulators”) OR TS = (“soluble guanylate stimulators”) OR TS = (“sGC activators”) OR TS = (“soluble guanylate cyclase activators”) OR TS = (“soluble guanylate activators”) OR TS = (“vericiguat”) OR TS = (“riociguat”) OR TS = (“praliciguat”) OR TS = (“cinaciguat”)OR TS = (“mosliciguat”) OR TS = (“avenciguat”) OR TS = (“runcaciguat”) OR TS = (“olinciguat”) OR TS = (“zagociguat”) OR TS = (“BAY-747”) OR TS = (“YC-1”) OR TS = (“BAY-412272”) OR TS = (“BAY41-8543”) OR TS = (“lificiguat”) OR TS = (“ataciguat”) OR TS = (“CFM-1571”) OR TS = (“A-350619”) OR TS = (“BAY 58-2667”) OR TS = (“HMR-1766”) OR TS = (“BAY 63-2521”) OR TS = (“IW-1973”) OR TS = (“K-5475”). Only the English papers published before 2024 with the type of article or review article were selected, and a total of 1,879 papers were retrieved for follow-up research. The specific process and filtering procedure are shown in Figure 1.

### 2.2 Data query and analysis

Data collection is primarily conducted through database searches. Directly query in WOSCC for the following information: publication volume, annual publication volume, citation frequency, citations per article, and the H index of the top 10 countries, institutions, authors, and journals, and so on. The H-index indicates that within a specific subject’s published papers, there are H papers with a citation frequency of at least H times (Hirsch, 2005; Alonso et al., 2009). Simultaneously, we also conducted a search for the annual citations of highly cited papers and the impact factors of journals. The Impact Factor (IF) is an internationally recognized index for evaluating journals. It was first proposed by Eugene Garfield, the founder of the American Institute for Scientific Information (ISI), in the 1960s (Garfield, 2006). In general, a high impact factor indicates that the academic influence of a journal is substantial and the quality of papers is relatively high.

The analysis of data primarily utilizes VOS viewer 1.6.20.0, CiteSpace 6.3R1 Advanced, and an online tool (WeShengXin) for visual analysis. The VOS viewer is capable of visualizing the critical points within a cluster and illustrating the collaboration between these points (van Eck and Waltman, 2010). Due to the advantages of simple operation and good visualization effect of this software, this study mainly uses it to conduct collaborative analysis of countries, authors or institutions, present the co-occurrence of keywords, and intuitively illustrate the co-citation relationship between authors, journals or references. CiteSpace is a robust tool for analyzing scientific literature, offering excellent visualization effects, strong operability, high interactivity, and a user-friendly interface (Chen, 2006). It has extensive applications in scientometrics and data visualization, making it an essential tool for researchers to analyze literature and construct knowledge maps. At the same time, because the software has multidimensional literature analysis, custom analysis function, efficient cluster analysis and other highlights, our researchers use it to generate keyword and reference keyword burst, reference burst, cluster analysis and time line cluster analysis. These analyses are employed to examine research hotspots, comprehend research trends, and assist researchers in understanding changes in research hotspots and trends over time. Weishengxin (https://www.bioinformatics.com.cn/) is a powerful, easy-to-operate, practical, efficient, and convenient online bioinformatics analysis and visualization cloud platform, which is a handy tool for scientific research workers. This online platform has gained the favor of many researchers due to its advantages of having numerous drawing templates and easy operation. In this analysis process, we mainly used it to visualize the world’s publication volume and display the annual publication volume of the top ten countries and the annual citation status of highly cited literature. R software is an open-source and powerful mathematical programming language that can be used for statistical analysis, data mining, machine learning, data visualization, and many other aspects. Especially its bibliometrix package is very convenient and comprehensive for bibliometric analysis.

## 3 Results

### 3.1 Results of publication volume

**FIGURE 2:**
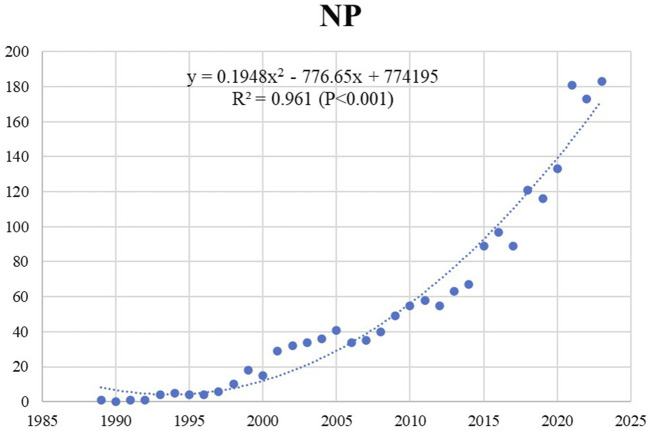
The scatter illustrating of annual publication volume (NP represents the number of annual publications).

We have analyzed 1,582 articles and 297 review articles that were published in English before the year of 2024. To comprehend the overall development trend of publications, we have created a scatter plot illustrating the annual publication volume (Figure 2) and indicated the trend line. It is apparent from the graph that there is a strong correlation between the annual publication volume and year of papers on sGC stimulators or activators, as indicated by a trend line with an R^2^ value of 0.961. Especially since riociguat became the first approved sGC stimulator in 2013, the research in this field has entered a stage of rapid development. The number of publications in the decade following 2013 is two times that of the years preceding 2013, accounting for nearly 66.5% of the total number of publications.

### 3.2 Distribution of countries and institutions

**FIGURE 3:**
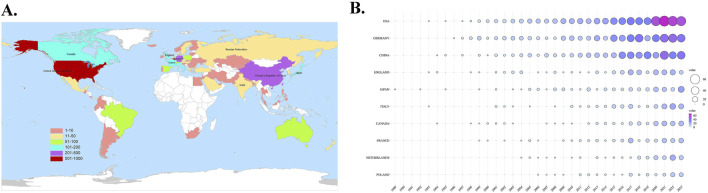
**(A)** The global distribution map of publication volume **(B)** The bubble chart of annual publication volume in the top 10 productive countries.

The researchers have conducted an analysis and organization of the top 10 productive countries/regions and co-authorship countries, which are listed in Table 1. It can be found that United States, Germany, China, England, Japan, Italy, Canada, France, Netherlands and Poland are among the top 10 countries in terms of publication volume. Germany is the country with the most frequent cooperation with other countries, publishing a total of 482 papers on sGC stimulants or activators, with a total link strength of 948. The United States cooperated with other countries slightly less frequently than Germany, ranking second. Although China and Japan have published a significant number of articles, their collaboration with other countries is not frequent. This suggests that there is a need for them to enhance their international cooperation in order to promote further development in this field. The researchers also used WeShengXin, an online tool, to draw a global distribution map of publication volume (Figure 3A) and a bubble chart of annual publication volume for the top 10 productive countries (Figure 3B). Figure 3A provides a rough representation of the publication status of countries worldwide. Different fill colors indicate varying publication volumes, with dark red indicating countries with a total publication volume exceeding 600. In Figure 3B, it is evident that the United States and Germany have significantly outpaced other countries in the annual number of publications, establishing themselves as key pioneers and leaders in this field. In addition, we can also see from the figure that China has been in a state of rapid development in this field in recent years.

**TABLE 1:** Top 10 productive countries/regions and co-authorship countries.

Rank	Country/Region	Count	Total citations	H-index	Rank	Co-authorship country	Count	Citations	Total link strength^1^
1	United States	637	20,385	73	1	Germany	482	23,559	948
2	Germany	482	20,489	79	2	United States	637	22,558	855
3	China	411	9942	53	3	England	159	7385	532
4	England	159	7107	46	4	France	111	6056	469
5	Japan	146	3241	32	5	Italy	128	6847	454
6	Italy	129	6632	36	6	Canada	113	5104	396
7	Canada	113	4891	31	7	Netherlands	86	4145	374
8	France	111	5837	40	8	Poland	63	3807	252
9	Netherlands	86	4029	27	9	Switzerland	51	3330	250
10	Poland	63	3686	24	10	Belgium	50	2488	244

The top 10 prolific institutions and co-authorship institutions are shown in Table 2. The institution with the highest number of publications is Bayer AG located in Germany, which has published a total of 255 papers on sGC stimulators or activators. At the same time, Bayer AG is also the institution that engages in the most frequent collaborations with others, thereby significantly advancing academic exchange and development in this field. In addition, seven of the top 10 prolific institutions belong to Germany. This indicates that Germany’s prosperous development in this field is closely related to its having more mature research institutions.

**TABLE 2:** Top 10 productive institutions and co-authorship institutions.

Rank	Productive institution	Count	H-index	Affiliated countries	Rank	Co-authorship institution	Affiliated countries
1	Bayer AG	255	65	Germany	1	Bayer AG	Germany
2	Bayer Healthcare Pharmaceuticals	126	57	Germany	2	University of Alberta	Canada
3	Hannover Medical School	88	29	Germany	3	Hannover Medical School	Germany
4	University of California System	80	33	United States	4	University of Giessen	Germany
5	Free University of Berlin	77	31	Germany	5	Duke University	United States
6	Assistance Publique Hopitaux Paris (APHP)	68	29	France	6	Inova Heart and Vasc Inst	United States
7	Institut National De La Sante Et De La Recherche Medicale (INSERM)	66	27	France	7	Merck and Co Inc.	United States
8	Berlin Institute of Health	64	26	Germany	8	Imperial College London	England
9	Charite Universitatsmedizin Berlin	64	26	Germany	9	German Ctr Lung Res Dzl	Germany
10	Humboldt University of Berlin	64	26	Germany	10	Duke National University of Singapore	Singapore

### 3.3 Distribution of authors

**FIGURE 4:**
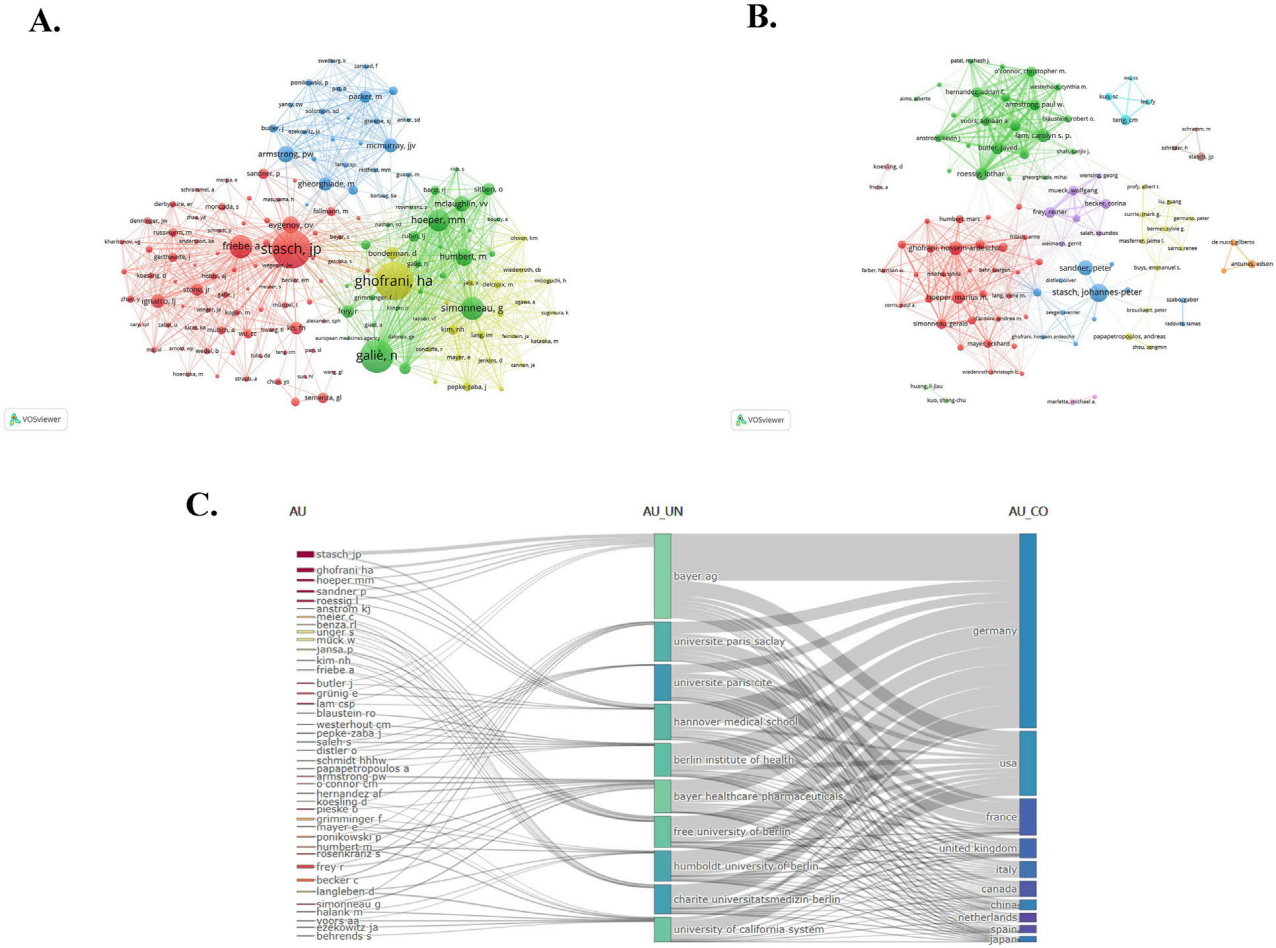
**(A)** VOS viewer network visualization of co-cited authors. Of the 33,777 co-cited authors, 150 had at least 50 citations. **(B)** Network visualization of co-authorship authors. Of the 8,968 authors, 100 had published at least 8 papers. **(C)** The Sankey map of the top 50 authors’ institutions and countries in terms of publication volume.

In this study, 8,968 authors participated in the publication of the papers we collected. The top 10 productive authors and co-cited authors are shown in Table 3. If several authors are cited concurrently in subsequent literature, they are considered to have a co-cited relationship. Researchers applied VOS viewer to visualize the network of co-cited authors (Figure 4A), including 150 authors that are cited at least 50 times. The same color represents the same cluster, while the size of the node indicates the cited frequencies. The line between two nodes signifies a co-cited relationship, with the thickness of the lines representing the degree of connection closeness. Professor Stasch, JP has published the highest number of papers, with a total of 86 papers. Following closely behind is Professor Sandner, P, who has published 45 papers, and Professor Hoeper, MM, who has published 41 papers. The most frequently cited author is Professor Stasch, JP, who has been cited 934 times and has a total link strength of 13,806. There are three authors who are part of both the top 10 productive authors and the top 10 co-cited authors, namely, Stasch, JP, Hoeper, MM, and Ghofrani, HA. This indicates that they not only produce a large number of articles but also have high-quality papers, which are generally recognized by the industry. Furthermore, we also conducted research on the collaborative relationships of the authors and created a network visualization of co-authorship authors (Figure 4B). It comprises 100 authors who have published at least 8 papers. The size of the nodes represents the frequency of cooperation, and the line between the two nodes signifies the cooperative relationship. It is evident that collaboration within the cluster occurs frequently, while collaboration between clusters is less common. At the same time, we have observed that Professor Stasch, JP and Professor Ghofrani, HA frequently collaborate with other authors and hold central positions within their respective clusters.

**TABLE 3:** Top 10 productive authors and co-cited authors.

Rank	Author	Count	Affiliated institutions/countries		Rank	Co-cited authors	Co-citations	Affiliated institutions/countries		Total link strength
1	Stasch, JP	86	Bayer AG	Germany	1	Stasch, JP	934	Bayer AG	Germany	13,806
2	Sandner, P	45	Bayer AG	Germany	2	Ghofrani, HA	918	Bayer AG	Germany	17,078
3	Hoeper, MM	41	Hannover Medical School	Germany	3	Galiè, N	774	University of Bologna	Italy	17,442
4	Ghofrani, A	35	Bayer AG	Germany	4	Friebe, A	492	University of Wurzburg	Germany	7298
5	Teng, CM	34	National Taiwan University	China	5	Simonneau,G	481	Universite Paris Saclay	France	11,542
6	Lam, CSP	33	National Heart Centre Singapore	Singapore	6	Hoeper, MM	457	Hannover Medical School	Germany	13,246
7	Roessig, L	31	Bayer AG	Germany	7	Humbert, M	336	Assistance Publique Hopitaux Paris (APHP)	France	9814
8	Kuo, SC	31	China Medical University Taiwan	China	8	Evgenov,OV	322	Harvard Medical School	United States	4649
9	Frey, R	28	Bayer AG	Germany	9	Armstrong,PW	320	University of Alberta	Canada	4187
10	Pieske, B	27	Berlin Institute of Health	Germany	10	Mclaughlin, VV	295	University of Michigan	United States	8052

Figure 4C is a sankey map of the top 50 authors’ institutions and countries in terms of publication volume, drawn by researchers using the R software bibliometrix package. Different nodes in the picture represent different authors, institutions, or countries, and lines in the picture represent relationships to which they belong. The width of the line represents the strength of the attribution, and the wider the line, the stronger the attribution. It can be found that the top ranked institutions and countries of these 50 authors are Bayer AG and Germany, respectively, which once again confirms the important position of Bayer AG and Germany in this field mentioned earlier.

### 3.4 Distribution of journals

**FIGURE 5:**
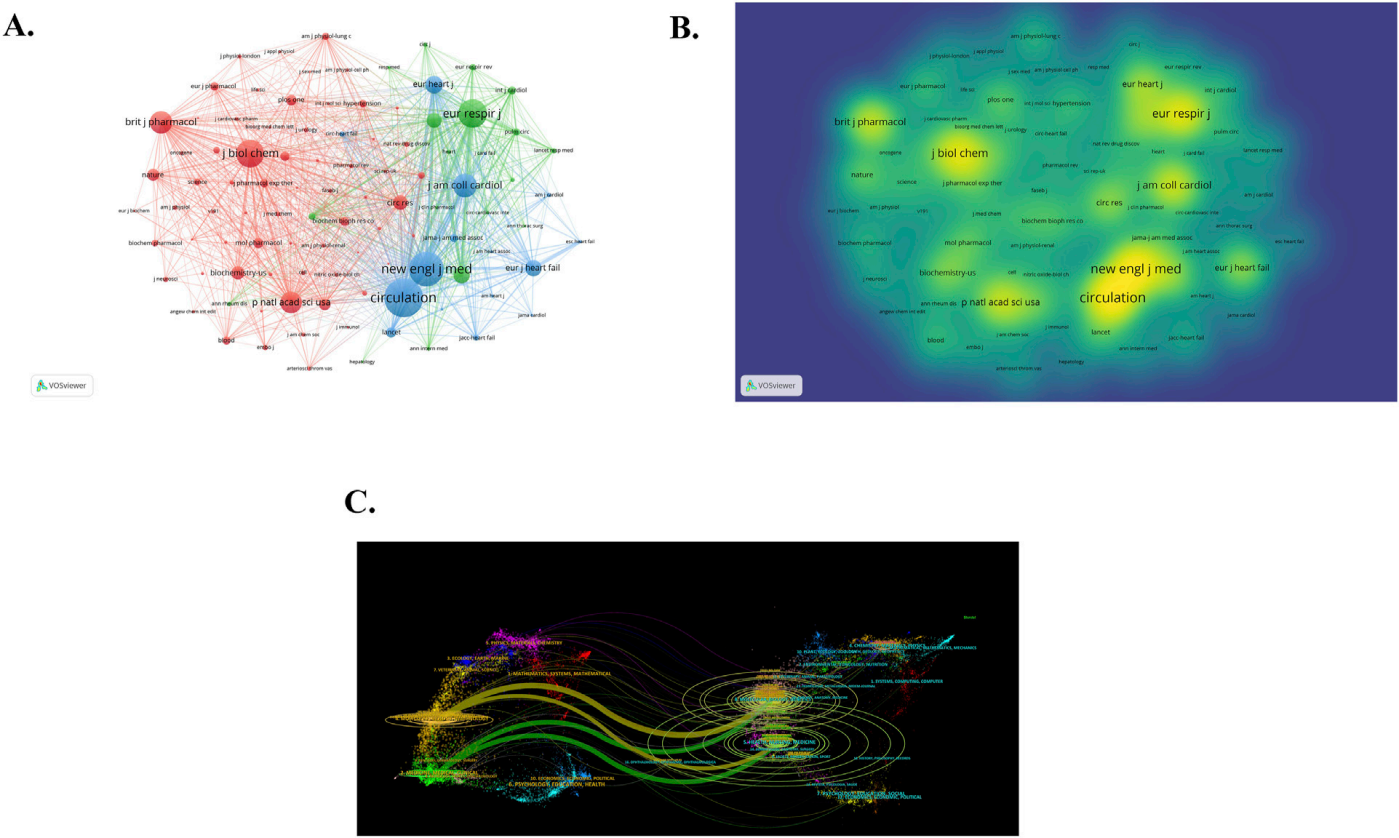
Analysis of co-cited journal **(A)** Network visualization of co-cited journals. **(B)** Density visualization of co-cited journals. Of the 6,281 co-cited journals, 100 had at least 140 citations. **(C)** The dual journal overlay map.

The top 10 productive journals and co-cited journals are listed in Table 4. The journal that ranks first in terms of publication volume is PULMONARY CIRCULATION (IF 2.2), with 52 articles published. The journal with the most citations is CIRCULATION, with an impact factor of 35.5 and total link strength of 184,395. It has been cited 3404 times, followed by NEW ENGL J MED (IF 96.2) and EUR RESPIR J (IF 16.6). It is easy to observe that most journals with highly cited frequency are distributed in Q1. The network visualization of co-cited journals is depicted in Figure 5A, encompassing 100 journals cited at least 140 times. The larger and more prominent points on the diagram represent several journals that are cited more frequently. Figure 5B is the density visualization of co-cited journals. The warmer the color and the larger the font, the higher the cited frequency. It is obvious from Figures 5A,B that CIRCULATION and NEW ENGL J MED are the most frequently cited journals. In addition, researchers also utilized CiteSpace software to create a dual journal overlay map (Figure 5C). The Double journal overlay map has the capability to generate new spatial relationships and attribute feature relationships through the superposition analysis of multi-layer data. This process allows for a more comprehensive understanding of the data and can reveal insights that may not be apparent when analyzing individual layers separately. Simultaneously, the double journal overlay map can uncover the flow of knowledge between disciplines and serve as a foundation for evaluating and selecting periodicals. The left part of Figure 5C pertains to the citing journals, which represent the knowledge frontier, while the right part depicts the cited journals, representing the knowledge base of the field. The different colors in the graph indicate different journal clusters, and the lines between the left and right sides signify citation relationships. From the image, it is apparent that “2 MEDICINE, MEDICAL, CLINICAL” and “4 MOLECULAR, BIOLOGY, IMMUNOLOGY” stand out as the most influential citing journal clusters in the knowledge frontier. In the knowledge base, the most influential cited journal clusters are the clusters marked as “5 HEALTH, NURSING, MEDICINE” and marked as “8 MOLECULAR, BIOLOGY, GENETICS”. The research findings published in these journals serve as the essential cornerstones of the field, playing a crucial role in its development and serving as necessary prerequisites for the smooth conduct of subsequent research. Also, in this study, there are four main citation paths, and each citing journal has multiple citation paths, showing an aggregation relationship with the cited journals.

**TABLE 4:** Top 10 prolific journals and co-cited journals.

Rank	Prolific journal	Count	H-index	JCR (2023)	If (2023)	Rank	Co-cited journal	Co-citations	Total link strength	JCR (2023)	If (2023)
1	PULM CIRC	52	14	Q2/Q3	2.2	14	CIRCULATION	3404	184,395	Q1	35.5
2	BRIT J PHARMACOL	49	26	Q1	6.8	26	NEW ENGL J MED	3020	143,343	Q1	96.2
3	EUR J PHARMACOL	35	16	Q1	4.2	16	EUR RESPIR J	2434	154,433	Q1	16.6
4	PLOS ONE	31	16	Q1	2.9	16	J BIOL CHEM	2312	76,145	Q2	4
5	J PHARMACOL EXP THER	26	16	Q2	3.1	16	J AM COLL CARDIOL	1951	112,991	Q1	21.7
6	J BIOL CHEM	23	22	Q2	4	22	BRIT J PHARMACOL	1852	63,314	Q1	6.4
7	NITRIC OXIDE-BIOL CH	21	13	Q2/Q3	3.2	13	PNAS	1736	61,127	Q1	9.4
8	BIOCHEM PHARMACOL	20	13	Q1	5.3	13	EUR HEART J	1393	86,048	Q1	37.6
9	BIOCHEMISTRY-US	20	18	Q3	2.9	18	CHEST	1287	85,904	Q1	9.5
10	INT J MOL SCI	20	7	Q1/Q2	4.9	7	EUR J HEART FAIL	1283	74,017	Q1	16.9

### 3.5 The results of the highly cited papers

**FIGURE 6:**
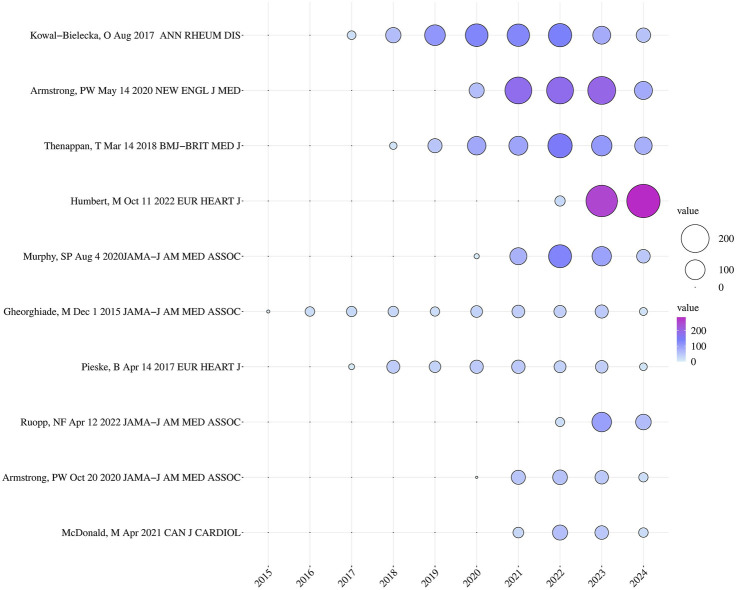
The bubble chart of the annual citation frequency of the top 10 highly cited papers.

Highly cited papers refer to papers published within the past 10 years that have been cited in the top 1% of the world rankings in the discipline. Our researchers have compiled the top ten highly cited papers in this field and listed them in Table 5. These 10 highly cited papers include 6 articles and 4 review articles. The top ranked highly cited paper is a review article, published by Kowal Bielecka, O et al. in ANN RHEUM DIS in 2017. This article suggests that the use of riociguat should be considered when treating systemic sclerosis (SSc) associated pulmonary arterial hypertension (PAH) (Kowal-Bielecka et al., 2017). So far, this paper has been cited 716 times. Figure 6 is a bubble chart of the annual citation frequency of highly cited papers ranked 10, drawn by researchers. The “2022 ESC/ERS Guidelines for the diagnosis and treatment of pulmonary hypertension, developed by the task force for the diagnosis and treatment of pulmonary hypertension of the European Society of Cardiology (ESC) and the European Respiratory Society (ERS), and endorsed by the International Society for Heart and Lung Transplantation (ISHLT) and the European Reference Network on rare respiratory diseases (ERN-LUNG)”, was published by Humbert, M et al. in the EUROPEAN HEART JOURNAL in 2022 (Humbert et al., 2022). It was explicitly stated that riociguat has a significant effect on chronic thromboembolic pulmonary hypertension (CTEPH), leading to a reduction in 6-min walk distance (6mwd) and a decrease in pulmonary vascular resistance by 31% compared with the control group. Upon publication, it immediately garnered significant attention from researchers and has been widely referenced due to its strong value as a reference. Meanwhile, the study “Vericiguat in Patients with Heart Failure and Reduced Ejection Fraction”, authored by Armstrong, PW et al. and published in the NEW ENGL J MED in 2020, has had a significant impact and has been frequently cited since its release. The authors demonstrated the clinical efficacy of vericiguat in patients with HFrEF, thus making a substantial contribution to the approval and launch of vericiguat in the market (Armstrong et al., 2020b). Besides, we also found that 4 out of these 10 highly cited papers were about the application and efficacy of riociguat in PAH or PH, while the remaining 6 focused on the application and efficacy of vericiguat in patients with HF, including heart failure with reduced ejection fraction (HFrEF) and heart failure with preserved ejection fraction (HFpEF). These are likely concrete manifestations of current research hotspots.

**TABLE 5:** Top 10 highly cited papers.

Rank	Highly cited papers	If (2023)	Total citations	Document type
1	Kowal-Bielecka, O August 2017, ANN RHEUM DIS	20.3	716	Review
2	Armstrong, PW 14 May 2020, NEW ENGL J MED	96.2	709	Article
3	Thenappan, T 14 Mar 2018, BMJ-BRIT MED J	93.6	574	Review
4	Humbert, M 11 Oct 2022, EUR HEART J	37.6	567	Article
5	Murphy, SP 4 Aug 2020, JAMA-J AM MED ASSOC	63.1	354	Review
6	Gheorghiade, M 1 Dec 2015, JAMA-J AM MED ASSOC	63.1	267	Article
7	Pieske, B 14 Apr 2017, EUR HEART J	37.6	260	Article
8	Ruopp, NF 12 Apr 2022, JAMA-J AM MED ASSOC	63.1	178	Review
9	Armstrong, PW 20 Oct 2020, JAMA-J AM MED ASSOC	63.1	171	Article
10	McDonald, M April 2021, CAN J CARDIOL	5.8	153	Article

### 3.6 The results of the references

We utilized VOS viewer as an analytical tool to conduct a co-cited reference analysis on all the papers involved in this study and have presented the top 5 co-cited references in Table 6. The article authored by Ghofrani, HA et al. in 2013 is the most significant reference within this particular field of study. It has been experimentally demonstrated in this article that riociguat can markedly improve motor capacity and pulmonary vascular resistance in patients with CTEPH (Ghofrani et al., 2013a). The second co-cited reference was a review published by Evgenov, OV et al., in 2006. The authors systematically elaborated on the fundamental information and clinical potential of sGC stimulators and activators in a comprehensive manner (Evgenov et al., 2006). The publication of this review greatly facilitates subsequent researchers in gaining a comprehensive understanding of sGC stimulators or activators. This will enhance convenience and efficiency for future research endeavors in this area. The paper about YC-1 published by Ko FN et al. in 1994 in BLOOD is the third ranked reference in this field discovered in this study. Its authors pointed out through experimental data that YC-1 is a sGC activator in rabbit platelets and found that it may have the potential to inhibit thrombosis (Ko et al., 1994). The paper “Soluble Guanylate Cyclase as an Emerging Therapeutic Target in Cardiopulmonary Disease” was published in CIRCULATION in 2001 by Stasch, JP et al., and is the fourth co-cited reference (Stasch et al., 2011). It has extremely profound significance and influence, and to this day it is still in a constantly cited state. The fifth co-cited reference was published by Galiè, N et al. in the journal of EUR HEART J in 2016. It is a highly authoritative diagnostic and treatment guideline for PH (Galiè et al., 2016). Although the guidelines have been updated (Humbert et al., 2022), there is no doubt about the beneficial efficacy of sGC stimulators for PH.

**TABLE 6:** The top 5 co-cited references.

Rank	Title	Author	Journal	DOI	Citations	Year
1	Riociguat for the Treatment of Chronic Thromboembolic Pulmonary Hypertension	Ghofrani, HA	NEW ENGL J MED	10.1056/nejmoa1209657	337	2013
2	NO-independent stimulators and activators of soluble guanylate cyclase: discovery and therapeutic potential	Evgenov,OV	NAT REV DRUG DISCOV	10.1038/nrd2038	245	2006
3	YC-1, a novel activator of platelet guanylate-cyclase	KO, FN	BLOOD	10.1182/blood.v84.12.4226. bloodjournal84124226	231	1994
4	NO-independent regulatory site on soluble guanylate cyclase	Stasch, JP	NATURE	10.1038/35065611	210	2001
5	2015 ESC/ERS Guidelines for the diagnosis and treatment of pulmonary hypertension The Joint Task Force for the Diagnosis and Treatment of Pulmonary Hypertension of the European Society of Cardiology (ESC) and the European Respiratory Society (ERS) Endorsed by: Association for European Paediatric and Congenital Cardiology (AEPC), International Society for Heart and Lung Transplantation (ISHLT)	Galiè, N	EUR HEART J	10.1093/eurheartj/ehv317	202	2016

Abbreviations (involved in the table section): APHP, assistance publique hopitaux paris; INSERM, institut national de la sante et de la recherche medicale; PULM CIRC, pulmonary circulation; BRIT J PHARMACOL, british journal of pharmacology; EUR J PHARMACOL, european journal of pharmacology; PLOS ONE, PLoS one; J PHARMACOL EXP THER, journal of pharmacology and experimental therapeutics; J BIOL CHEM, journal of biological chemistry; NITRIC OXIDE-BIOL CH, Nitric Oxide-Biology and Chemistry; BIOCHEM PHARMACOL, biochemical pharmacology; BIOCHEMISTRY-US, biochemistry; INT J MOL SCI, international journal of molecular sciences; NEW ENGL J MED, new england journal of medicine; EUR RESPIR J, european respiratory journal; J BIOL CHEM, journal of biological chemistry; J AM COLL, CARDIOL, journal of the american college of cardiology; BRIT J PHARMACOL, british journal of pharmacology; EUR HEART J, european heart journal; EUR J HEART FAIL, European Journal of Heart Failure; ANN RHEUM DIS, annals of the rheumatic diseases; BMJ-BRIT MED J, BMJ-British Medical Journal; JAMA-J AM MED ASSOC, JAMA-Journal of The American Medical Association; NAT REV DRUG DISCOV, nature reviews drug discovery; CAN J CARDIOL, canadian journal of cardiology.

**FIGURE 7:**
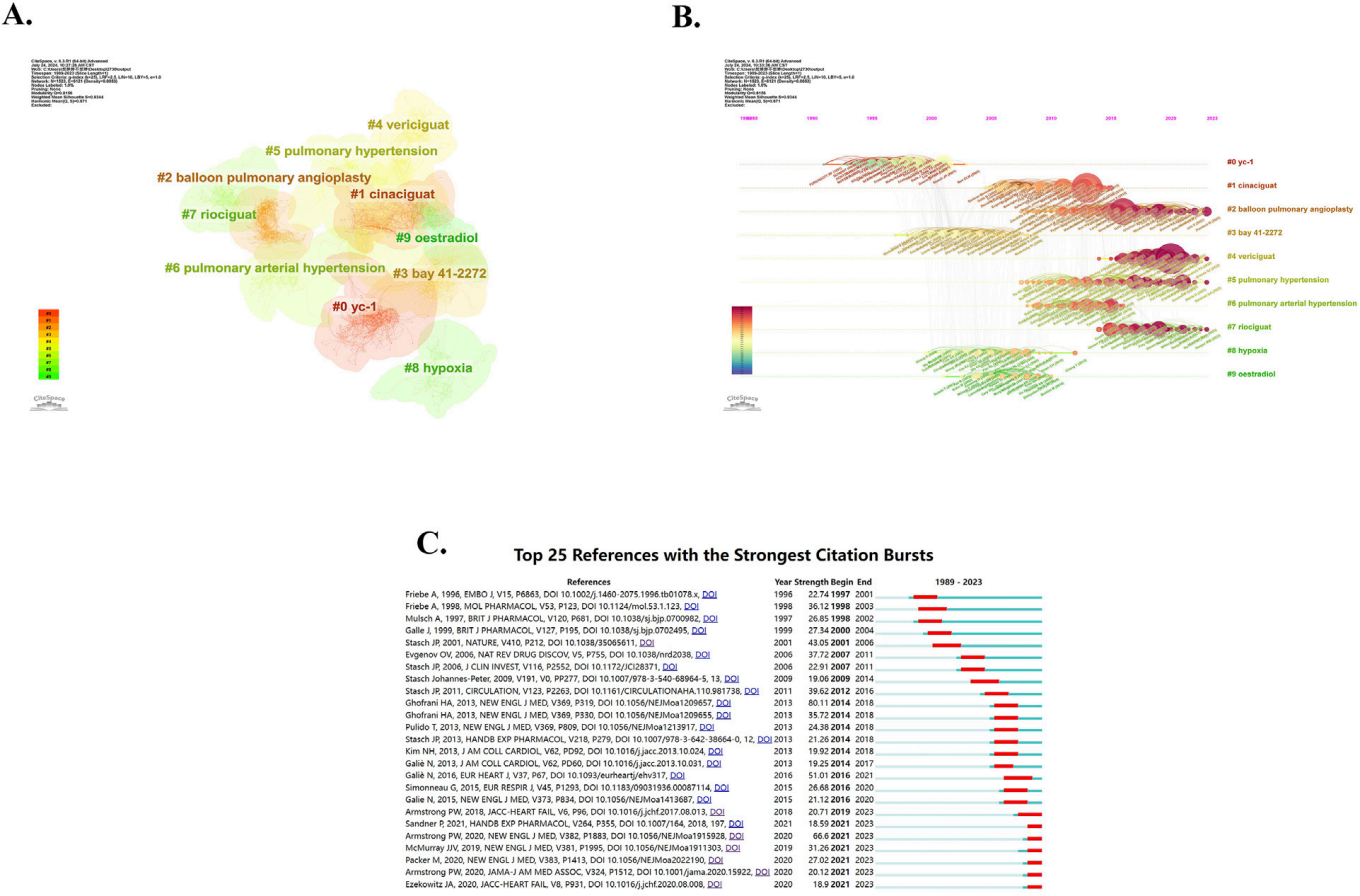
Analysis of reference. **(A)** Cluster analysis of reference. **(B)** Timeline view of co-cited reference. **(C)** The top 25 references with the strongest citation bursts.

We also created the co-cited reference cluster analysis using CiteSpace, shown in Figure 7A. The Modularity Q of this cluster analysis is high, which is 0.8156, and the Weighted Mean Silhouette S is 0.9344, indicating that the cluster structure is significant and convincing. The references are divided into 22 clusters (cluster #0-#21), and we have selected the top 10 of them for visualization. Different colors represent different clusters, and the size of the clusters indicates the volume of content contained within each cluster. Cluster #0, labeled as “yc-1”, is the largest cluster composed of 174 references, followed by cluster #1, labeled as “cinaciguat”, which contains 141 references. Cluster #2, named “balloon pulmonary angioplasty”, contains 139 references. The cluster #3, named “bay 41-2272”, contains 139 references; and cluster #4, named “vericiguat”, contains 135 references. The cluster #5, “pulmonary hypertension”, contains a total of 110 references. The next cluster #6 (including 88 references), cluster #7 (including 74 references), and cluster #8 (including 63 references), were respectively named “pulmonary arterial hypertension”, “riociguat” and “hypoxia”, while the cluster #9 is labeled as “oestradiol” and contains 42 references. Figure 7B is a timeline view of co-cited references, which effectively demonstrates both the outcomes of the clusters and the temporal evolution. Each data point on the graph represents a reference, while the curves between the points depict the co-cited relationships. The size of each data point reflects its cited frequency. It can be found that cluster #2, cluster #4, cluster #5, and cluster #7 emerge in recent years and have high citation frequency. The top 25 references with the strongest citation bursts are shown in Figure 7C. The period between the Begin and End years signifies the peak influence of the reference. The dark blue represents the year of publication, while red indicates the duration of impact. Among them, the strongest reference for explosive intensity is “Riociguat for the Treatment of Chronic Thromboembolic Pulmonary Hypertension”, published by Ghofrani, HA et al.in NEW ENGL J MED in 2013 (Ghofrani et al., 2013a), with a citation strength of 80.11. It has been highly influential and significant in promoting riociguat to become the first approved sGC stimulator. The last few references on the Figure are the ones that still have highly influence so far. They are the “A Multicenter, Randomized, Double-Blind, Placebo-Controlled Trial of the Efficacy and Safety of the Oral Soluble Guanylate Cyclase Stimulator The VICTORIA Trial”, published by Armstrong PW et al., in 2018 in JACC-HEART FAILURE (Armstrong et al., 2018), “Soluble Guanylate Cyclase Stimulators and Activators.” authored by Sandner, P in 2021in HANDB EXP PHARMACOL (Sandner et al., 2021b), “Vericiguat in Patients with Heart Failure and Reduced Ejection Fraction”, published by Armstrong PW et al. in 2020 in NEW ENGL J MED (Armstrong et al., 2020b), “Dapagliflozin in Patients with Heart Failure and Reduced Ejection Fraction”, published by McMUrray JJV et al. in NEW ENGL J MED in 2019 (McMurray et al., 2019), “Cardiovascular and Renal Outcomes with Empagliflozin in Heart Failure”, published by Packer M et al., in 2020 in NEW ENGL J MED (Packer et al., 2020), “Effect of Vericiguat vs. Placebo on Quality of Life in Patients With Heart Failure and Preserved Ejection Fraction The VITALITY-HFpEF Randomized Clinical Trial”, which is published by Armstrong PW et al., in 2020 in JAMA-J AM MED ASSOC (Armstrong et al., 2020a), and “N-Terminal Pro-B-Type Natriuretic Peptide and Clinical Outcomes”, published by Ezekowitz JA et al. in JACC-HEART FAIL in 2020 (Ezekowitz et al., 2020). These findings suggest that current research is primarily focused on the clinical effectiveness and impact of several emerging drugs, such as riociguat and vericiguat. Specifically, there is a particular emphasis on evaluating the efficacy of sGC stimulators or activators in addressing pulmonary hypertension (PH) and heart failure (HF). This focus may be attributed to the successful listing of two drugs, riociguat in 2013 and vericiguat in 2021.

### 3.7 The results of the keywords

Keywords are core words or phrases in a particular field or topic that describe, summarize, or identify that field or topic. The change of keywords often represents a change in the research trend of a field. In the course of this analysis, the researchers mainly used three different analysis software for research and visualization, in order to eliminate the accidental errors that can occur in a single analysis software.

**FIGURE 8:**
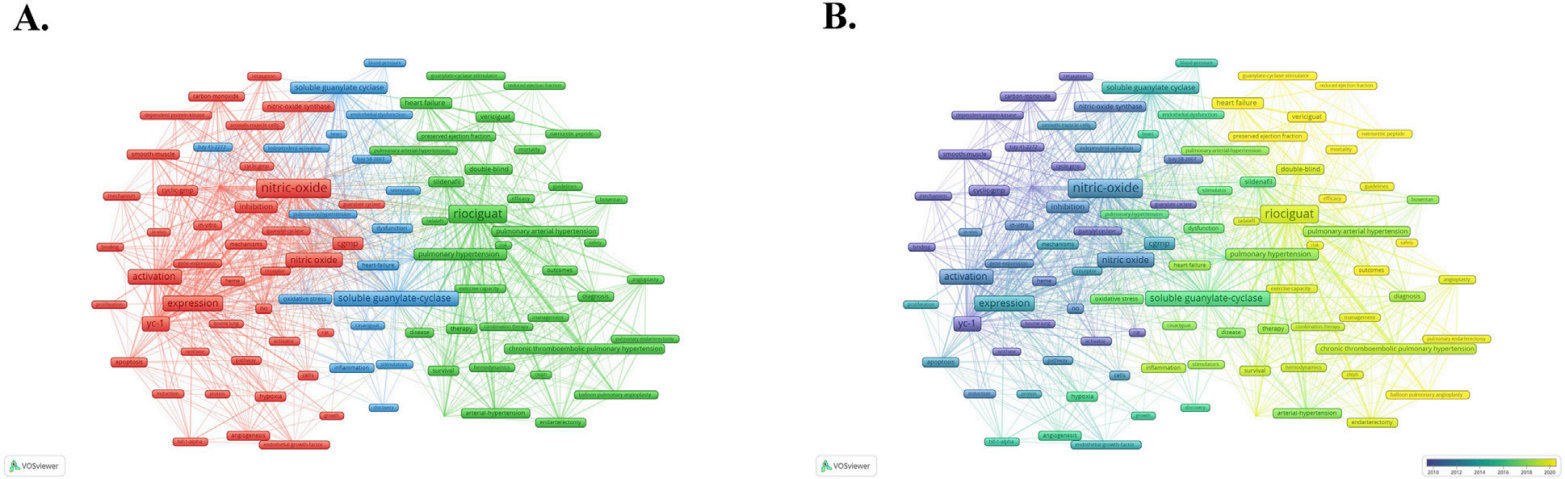
VOS viewer analysis of keywords. **(A)** Network visualization of keywords. **(B)** Overlay visualization of keywords. Of the 6,883 keywords, 100 had at least 30 occurrences.

The co-occurrence network visualization of keywords, created using VOS viewer, is shown in Figure 8A. A total of 6,883 keywords appeared in this study. The network visualization includes 100 keywords that appeared at least 30 times. They are categorized into 3 clusters: red is the first cluster containing 43 keywords, green is the second cluster containing 37 keywords and blue is the third cluster containing 20 keywords. The node size indicates the occurrence frequency of keywords, while the connections between nodes represent co-occurrence relationships. In the overlay visualization of keywords (Figure 8B), the node color represents the average year in which the clustering keyword appeared. The warmer node color indicates a later appearance of the keyword. As observed in Figure 8, the keywords “nitric oxide”, “expression”, “yc-1” “cyclic-gmp” are the earlier appearing and influential terms, while “riociguat”, “chronic thromboembolic pulmonary hypertension” and “pulmonary hypertension” are the keywords that appear later and with the higher frequency of occurrence. And “vericiguat”, “heart failure” and “mortality” have emerged as newly prominent keywords in the field.

**FIGURE 9:**
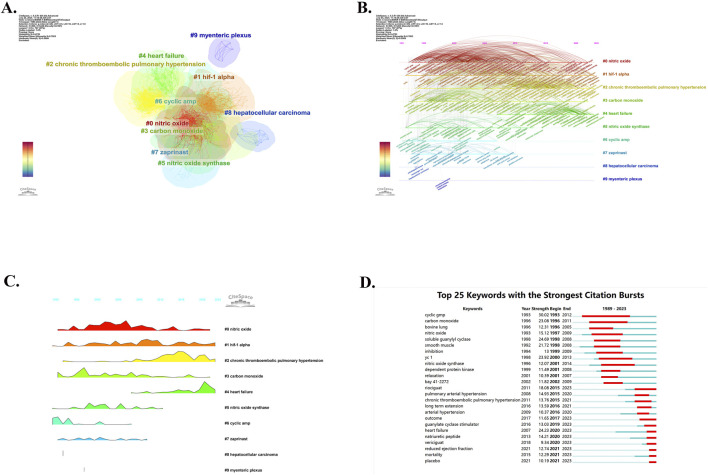
CiteSpace analysis of keywords. **(A)** Cluster analysis of keywords. **(B)** Timeline view of keywords. **(C)** Landscape view of keywords. **(D)** The top 25 keywords with the strongest citation bursts.

In addition to using VOS viewer for keyword co-occurrence analysis, the researchers also used CiteSpace software for keyword cluster analysis and visualized the cluster view (Figure 9A) and timeline view (Figure 9B). All of the keywords were divided into 12 clusters in cluster analysis (cluster #0-#11) with the Modularity Q of 0.4753 and the Weighted Mean Silhouette S of 0.7803. Only the top 10 keyword clusters are visualized in these figures. Cluster #0, labeled “nitric oxide”, contains 168 keywords, which is the largest and earliest keyword cluster. However, the occurrence frequency of keywords in this cluster has decreased in recent years. Cluster #1 is named “hif-1 alpha” and contains 146 keywords. Cluster #2, named “chronic thromboembolic pulmonary hypertension”, contains 142 keywords. The cluster #3, labeled “carbon monoxide”, contains 111 keywords. Cluster #4, “heart failure”, contains 73 keywords. Cluster #5, labeled “nitric oxide synthase”, contains 52 keywords, and cluster #6, which was named “cyclic amp”, contains 43 keywords. The following cluster #7 “zaprinast”, cluster #8 “hepatocellular carcinoma”, and cluster #9 “myenteric plexus” contain 30 keywords, 9 keywords, and 7 keywords, respectively. We also developed a landscape view of keywords (Figure 9C), which provides a clearer and more intuitive presentation of the appearance time and frequency of keywords. The height of the peaks indicates the frequency of occurrence of keywords. It can be found that cluster #0, cluster #1, cluster #3 and cluster #6 are the earliest keywords clusters, playing a vital role in developing this area. Cluster #2, named “chronic thromboembolic pulmonary hypertension” and Cluster #4, named “heart failure”, have exhibited a higher frequency of occurrence in recent years. The remaining cluster #5, cluster #7, cluster #8, and cluster #9 have hardly been seen in recent years, which seems to mean that research related to them is obsolete in the field. In addition, we also used CiteSpace to highlight the top 25 keywords with the strongest citation bursts (Figure 9D) so as to gain a better understanding of research hotspots and grasp development trends more effectively. It can be found that the top 1 keyword with the strongest CBs is “cyclic gmp”, with a strength of 30.02, which is the most influential keyword from 1993 to 2012. While “riociguat”, “outcome”, “guanylate cyclase stimulator”, “heart failure”, “natriuretic peptide”, “vericiguat”, “reduced ejection fraction”, “mortality” and “placebo” are the keywords that still have significant influence today.

The “bibliometrix” package of R software is a powerful toolkit developed and maintained by Massimo Aria and Corrado Cuccurullo for bibliometric analysis, which enables multidimensional analysis of selected literature (Aria and Cuccurullo, 2017). Supplementary Figure S1A is the keyword tree view of the top 50 keywords that the researchers plotted using the R package. The figure shows the frequency and percentage of each of these 50 keywords. The larger the area of the square where the keyword is located, the higher the frequency of the keyword. It can be clearly seen that the keywords “nitric oxide”, “expression” and “activation” are the top three keywords in the frequency of occurrence, which is consistent with the above analysis results. The Sankey plot (Supplementary Figure S1B) clearly shows the flow of keywords within different time slices, reflecting the changing trend of research focus in this field. It can be observed that research on nitric oxide has been ongoing throughout the entire development process of this field. At the same time, the researchers also analyzed the keyword clustering in the fifth time slice (2017-2024) in detail on the basis of Supplementary Figure S1B, and visualized it in Supplementary Figure S1C. Supplementary Figure S1C contains four keyword clusters in total, and they are distributed in four quadrants. Clustering located in the first quadrant (related to research on nitric oxide, expression, and activation) has a high level of development and relevance, and can be considered as a key research direction in this field (Motor Themes). The cluster named “guanylate cyclase simulator, natriuretic peptide, reduced ejection fraction” is distributed in the second quadrant and has a high level of development in recent years but low correlation. It can be considered as a niche research direction in this field (Niche Themes). Keyword clustering located in the third quadrant has a low degree of development and relevance, and is likely to be emerging or declining Themes in this field. Clustering in the fourth quadrant is likely to be the basic research in this field, because this keyword clustering has a high degree of relevance but a low degree of development for the present. This indicates that for the present time slice, the research on riociguat, soluble guanylate-cyclase, double-blind and so on is likely to be a hot research topic before it has important research value for the present research.

### 3.8 Scopus database validation

To verify the accuracy of the above analysis results, the researchers also used the same search terms in Elsevier’s Scopus database for paper search screening and analysis verification. In this database, we retrieved a total of 3,037 papers or reviews published in English between 1989 and 2023. According to the annual publication volume obtained from the database, the number of papers published in this field is significantly correlated with the year (R^2^ = 0.9536), which is very consistent with the research results of WoSCC data (Supplementary Figure S2). At the national level, the analysis results of both databases are highly consistent in terms of publication volume (Supplementary Figure S3) and cooperation (Supplementary Figure S4). The United States (USA), Germany and China are the top three countries in this field, especially the United States, whose number of publications is much higher than other countries, and is an important promoter of the development of this field. At the same time, Germany and the United States (USA) are also the countries that cooperate most closely with other countries, making great contributions to the exchange and collision of knowledge in this field. Supplementary Figure S5 and Supplementary Figure S6 are the author publication volume network views and co cited author network views visualized by researchers using VOS viewer based on the Scopus database.

Reference analysis based on the Scopus database are shown in Supplementary Figures S7-S10. Supplementary Figure S7 shows the clustering of references analyzed using CiteSpace software. The Modularity Q value of this clustering is 0.8595 and the Weighted Mean Silhouette S value is 0.9145, indicating that the classification of this clustering is very reasonable and the structure is very stable. The figure displays a total of 9 clusters from the top 10 reference clusters (cluster labeled #9 was not displayed due to insufficient content). Supplementary Figure S8 is a peak view corresponding to the reference clustering in Supplementary Figure S7. It can be observed that the cluster labeled #3 “yc-1” is the earliest reference clustering in this field, and has rarely appeared in recent years. Several reference clusters labeled as #1 “pulmonary arterial hypertension”, #2 “heart failure”, #4 “vericiguat”, #6 “chronic thromboembolic pulmonary hypertension”, and # 9 “worrying heart failure” still have a certain frequency of occurrence, indicating that research related to them is likely to be a current research hotspot. Supplementary Figure S9 and Supplementary Figure S10 respectively show the time zone distribution of the references (the references with high influence are marked in the figure) and the top 25 references with the strongest citation bursts. Comparing the reference studies based on the WoSCC database in the previous section, it is not difficult to find that although there are slight differences in the research results of the two databases, overall they show the same development trend and direction.

Supplementary Figures S11, S12 are the co-occurrence network visualization and overlay visualization of author keywords drawn by VOS viewer software based on the search and screening of Scopus database. According to the figure drawn this time, it is very obvious that, “pulmonary hypertension”, “pulmonary arterial hypertension”, “nitric oxide”, “soluble guanylate cyclase” and “heart failure” are the most frequently used keywords in this field. Especially the keyword of “heart failure”, it has emerged in recent years and has a high frequency of occurrence. In addition to using the VOS viewer software, the researchers also used the CiteSpace software to perform a more comprehensive analysis of keywords. The timeline view of keyword clustering analysis is visualized in Supplementary Figure S13. In this analysis process, all keywords were divided into 11 clusters (with a Modularity Q value of 0.5847 and the Weighted Mean Silhouette S value of 0.8524). According to the visualization result, we find that the #0 marker labeled “soluble guanylyl cyclase” is the earliest keyword cluster in the field, and its occurrence frequency has decreased in recent years. The clusters named #8 “yc-1 activation”, #9 “drug administration”, and #10 “human erythrocyte” have hardly appeared in recent years, especially the cluster named #10 “human erythrocyte” which has not appeared for nearly 20 years. The remaining clusters have a certain frequency in recent years, which indicates that the current research focus is likely to focus on “pulmonary arterial hypertension”, “chronic thromboembolic pulmonary”, “heart failure”, “pulmonary angioplasty”, “systemic sclerosis”, “cardiovascular diseases” and “antiplatelet activity”. Supplementary Figure S14 reveals the top 25 keywords with the strongest citation bursts (sorted by starting year). As demonstrated in the figure, the three keywords (“Heart failure with reduced ejection fraction”, “hospitalization”, and “vericiguat”) continue to exert significant influence in contemporary research.

In summary, researchers found that although Scopus and WoSCC databases were slightly different in terms of analysis results, they were very consistent and compatible in general. This situation to some extent proves the accuracy of our research and reduces the occurrence of errors.

## 4 Discussion

### 4.1 Global trend

We collected a total of 1,879 papers for research spanning from 1989 to 2023. In the top 10 productive countries/regions, the United States occupies first place with 637 published papers, and Germany ranks second with 482 published papers. They contribute to 60% of the total number of publications, surpassing other countries worldwide by a large margin. There are seven institutions in the top 10 productive institutions belonging to Germany. Germany and the United States also rank first and second respectively among the top 10 countries with the highest collaboration frequency. In addition, seven of the top 10 productive authors are from Germany. Meanwhile, four of the top 10 co-cited authors are from Germany and two are from the United States. The aforementioned statement implies that in the field of research on sGC stimulators or activators, Germany and the United States have consistently held a dominant position, played a pivotal role, and served as pioneers. With their forward-thinking strategic vision, innovative scientific and technological prowess, and astute awareness of industry trends, they are spearheading the development direction of the field and setting a precedent for other nations. BAYER AG, located in Germany, is the most productive institution in this research field and has made a significant contribution to the field of research. Furthermore, several emerging drugs on the market have been developed by BAYER AG. The journal PULM CIRC (IF 2.2) has published the most papers in this field, with 52 papers. CIRCULATION (IF 35.5) was the most frequently cited journal in the study, with 3,404 citations. J BIOL CHEM (IF 4) is not only a journal with more published papers in this field, but also a journal with higher citation frequency in this study. Four of the top 10 highly cited papers were published in JAMA-J AM MED ASSOC (IF 63.1) and two in EUR HEART J (IF 37.6). This situation exemplifies the professionalism and industry recognition of high-quality journals. The papers published in these journals often wield significant influence and can assist researchers in gaining a deeper understanding of extensive research findings within a particular field.

### 4.2 Analysis of authors

Professor Stasch, JP is the most prolific author in the field and ranks first among the top 10 co-cited authors. He is a distinguished researcher with expertise spanning various fields, including pharmacology, cardiovascular system, respiratory system, biochemistry and molecular biology, and cell biology. Professor Stasch has played a significant role in advancing research on sGC stimulators or activators. In 1997, Professor Stasch, JP with his partners, through a large number of experiments, showed that YC-1 is a direct ionizing activator of sGC NO-independent and O_2_− sensitivity, and it can play vasodilatory effects by increasing the body’s cGMP (Mulsch et al., 1997). In 2001, Professor Stasch, JP pointed out a novel compound capable of effectively stimulating sGC independent of the NO mechanism, suggesting a new potential for cardiovascular disease treatment (Stasch et al., 2001). In 2006, Professor Stasch, JP and his colleagues noted that compounds which activate sGC in a manner independent of nitric oxide (NO) offer significant therapeutic benefits (Evgenov et al., 2006). In 2011, he suggested that soluble guanylate cyclase could serve as a novel therapeutic target for cardiopulmonary diseases. This suggestion has significantly advanced the development of treatments for cardiopulmonary diseases (Stasch et al., 2011). In recent years, Professor Stasch, JP has been conducting research on a novel compound of sGC stimulator, BAY-747. It has the opportunity to become a research hotspot in the future.

Professor Ghofrani, HA is the author with the second most cited frequency, and his research focus on the cardiovascular and respiratory systems, particularly on pulmonary hypertension (PH). In 2006, he participated in the publishing “Activation of soluble guanylate cyclase reverses experimental pulmonary hypertension and vascular remodeling”, which illustrated that bay41-2272 and bay58-2627 could significantly reverse pulmonary vascular remodeling in rats with severe pulmonary hypertension induced by monocrotaline injection (Dumitrascu et al., 2006). In 2010, Professor Ghofrani, HA and his collaborators conducted a 12-week Phase II clinical controlled trial. Their preliminary findings demonstrated that riociguat could enhance the exercise ability and alleviate clinical symptoms of chronic thromboembolic pulmonary hypertension (CTEPH) or pulmonary arterial hypertension (PAH) (Ghofrani et al., 2010). In particular, the “Riociguat for the Treatment of Chronic Thromboembolic Pulmonary Hypertension”, published in 2013 (Ghofrani et al., 2013a), has garnered significant attention from researchers and holds a pivotal position in this field. Professor Ghofrani, HA, has participated in and published a total of 683 papers so far, with a personal H-index of 95. It can be said that he is a scholar with great output and influence.

### 4.3 Analysis of research foundation

The study of mechanisms is a crucial prerequisite for the development of any field, as it can unveil the essence and internal connections of a subject. In the case of sGC, which serves as the target of sGC stimulators or activators, studying its physiological action and mechanism is an indispensable research foundation in this particular field.

In 1969, White, AA et al. detected sGC in mammals (Schultz et al., 1969; White and Aurbach, 1969). Of course, the presence of sGC was not detected by accident or whim, but after researchers first discovered cAMP in 1958, researchers began to study purposefully, thus discovering the existence of sGC. Around the 1990s, there was a general understanding of the signal transduction pathway of NO-sGC-cGMP. At that time, researchers discovered that the activation of sGC could increase the concentration of cGMP in organisms. Acting as a second messenger, cGMP can then target specific receptors and induce a series of subsequent physiological effects. This is the most important pathway for sGC to function (Denninger and Marletta, 1999). Additionally, the sGC also appears to have an inhibitory effect on the TGF-β signaling pathway, thereby carrying out additional functions (Beyer et al., 2015; Zenzmaier et al., 2015; Sandner and Stasch, 2017). These two pathways of action of sGC are the most fundamental research prerequisites in this field, as well as the fundamental foothold and important basis for research in this field.

Hypoxia is an important pathophysiological state in the body, which can inhibit the activity of sGC directly or indirectly. And the sGC activators or stimulators can effectively counteract the pathological effects of hypoxia by directly activating or enhancing NO signaling pathways. This provides strong research support for the development of subsequent drugs and the treatment of diseases. Estradiol enhances the efficacy of the sGC activator or stimulator by directly activating the sGC and the antioxidant mechanism. In the treatment of cardiovascular diseases and tumors, the combination of estradiol and sGC activators shows multi-target modulation and signal amplification effects, which has potential therapeutic advantages.

### 4.4 Analysis of research hotspots

Based on the analysis of the aforementioned research findings, it is our belief that the current focal points of research in this field primarily revolve around the development of various pharmaceuticals and the investigation into their clinical effectiveness and safety.

#### 4.4.1 Riociguat (BAY 63-2521)

Research on riociguat has been a prominent focus in this field, particularly due to its distinction as the first sGC stimulator to receive marketing approval in 2013.This highlights that any newly emerging drug needs to undergo a considerable number of clinical trials before being marketed, and its clinical efficacy and adverse reactions also need to be evaluated after being marketed.

In 2009, Grimminger, F et al. first described the application of riociguat in patients with PH and pointed out that riociguat has good tolerance (Grimminger et al., 2009). In the same year, Mittendorf, J et al. discovered that riociguat exhibited favorable pharmacokinetics and demonstrated the ability to impact the hemodynamics and motor capacity of patients with PH (Mittendorf et al., 2009). Subsequent clinical trials have demonstrated that riociguat has a beneficial impact on patients with PH while maintaining a high level of safety. (Ghofrani et al., 2010; Bonderman et al., 2013; Ghofrani et al., 2013a; Hoeper et al., 2013). Especially in patients with CTEPH, riociguat has shown excellent clinical efficacy and safety (Simonneau et al., 2015; Ghofrani et al., 2021). So far, research on riociguat is still ongoing. Researchers have confirmed that the combination of riociguat and interventional balloon angioplasty can potentially benefit patients with CTEPH who cannot undergo surgery, improving their 5-year survival rate (Jaïs et al., 2022; Wiedenroth et al., 2023). Riociguat can also be used for the treatment of pulmonary hypertension associated with connective tissue disease (CTD-PAH). While there is currently no conclusive evidence supporting the specific use of riociguat for systemic sclerosis-associated pulmonary hypertension, it should be considered as a potential treatment option for this condition (Kowal-Bielecka et al., 2017). In addition, in a 1-year double-blind controlled trial, riociguat demonstrated clinical efficacy in preventing clinical deterioration of sarcoidosis related pulmonary hypertension and improving exercise ability, and no major adverse events have been detected (Baughman et al., 2022). However, the researchers noted that riociguat does not appear to be as safe as currently believed, and it has been reported in the U.S. Food and Drug Administration Adverse Event Reporting System with more bleeding and low blood pressure (Patel et al., 2022). And a subsequent hemodynamic trial also showed that compared to placebo, riociguat led to more patients withdrawing due to adverse events (Dachs et al., 2022). The research on the security of riociguat seems to have unclear results so far, and further analysis and verification are still needed in subsequent projects.

#### 4.4.2 Vericiguat (BAY-1021189)

Vericiguat is a novel soluble guanylate cyclase stimulator that can directly stimulate sGC to enhance its sensitivity to NO. It has been primarily developed for the treatment of chronic heart failure (Follmann et al., 2017) and was approved for market by the FDA in 2021 (Markham and Duggan, 2021). Vericiguat has been well tolerated in numerous controlled clinical trials since its discovery (Boettcher et al., 2021). For patients with HFrEF, the change in NT-pro BNP in this group was not statistically significant when treated with vericiguat for 12 weeks (Gheorghiade et al., 2015). In long-term, randomized, double-blind controlled trials, vericiguat has demonstrated significant clinical efficacy by reducing cardiovascular mortality and the incidence of first hospitalization due to heart failure in patients with HFrEF (Armstrong et al., 2020b; Lombardi et al., 2021). Some researchers believe that vericiguat will be likely to become the fifth pillar of treatment for patients with HFrEF (Spadafora et al., 2024). Also, current research has indicated that vericiguat appears to have the potential to enhance cardiac function in patients with HFrEF (Suzuki et al., 2024). For patients with heart failure with preserved ejection fraction (HFpEF), the clinical efficacy of vericiguat does not seem to have reached a consensus at present. In 2017, Pieske et al. conducted a SOCRATES-PRESERVED study and reported that while 12 weeks of vericiguat treatment did not lead to improvements in NT-pro BNP and LAV for HFpEF patients, it did show potential for enhancing their quality of life (Pieske et al., 2017). However, a randomized clinical trial in 2020 found that treatment with doses of vericiguat at 15 mg/day or 10 mg/day for 24 weeks did not lead to improvement in physical restriction scores (PLS) for patients with HFpEF (Armstrong et al., 2020a). In a recent review of sGC stimulators, the authors have highlighted that the use of riociguat not only fails to yield significant positive effects on patients with HFpEF but may also result in drug-related adverse events (Zhao et al., 2024). The efficacy of vericiguat in patients with HFpEF seems to need further investigation (Jia et al., 2022).

#### 4.4.3 Other drugs

Cinaciguat (BAY 58-2667) is a first-generation sGC activator with potential therapeutic effects for acute decompensated heart failure (ADHF) (Lapp et al., 2009). Numerous studies have shown that cinaciguat can improve cardiac load in patients with ADHF, but high doses of cinaciguat seem to lead to the occurrence of hypotension (Erdmann et al., 2013). In addition, researchers have found that the drug appears to have a protective effect against ischemia-reperfusion injury (Salloum et al., 2012), as well as anti-platelet and anti-vascular remodeling effects (Hirschberg et al., 2013). Subsequently, based on the vasodilation and anti-remodeling effects of the drug on hypertension, some scholars found through animal experiments that cinaciguat can prevent the closure of the ductus arteriosus (DA) in mice after birth, providing new insights into the patency of the ductus arteriosus (DA) (Hung et al., 2021). In recent years, cinaciguat has been found to be effective in alleviating intestinal flora disorders and metabolic disorders induced by high fat diet, which indicates that mixed hyperlipidemia is likely to be a new therapeutic direction of this drug (Jia et al., 2023).

Praliciguat (IW-1973) is an sGC stimulator developed by Ironwood Pharmaceuticals. Currently, it is undergoing phase II clinical trials to assess its efficacy in patients with diabetic nephropathy (DN)and HFpEF (Shea et al., 2020). A study conducted in 2020 has indicated that praliciguat does not appear to have a significant therapeutic effect on patients with HFpEF (Udelson et al., 2020). In the treatment of diabetic nephropathy (DN), although no definitive conclusions have been reached, a number of experimental results support further research and exploration of praliciguat (Hanrahan et al., 2020; Hanrahan et al., 2021). In addition, a 2019 study conducted by Hall, KC et al. confirmed the potential of praliciguat to act as an anti-inflammatory and anti-fibrotic agent. The study also noted that praliciguat shows promise in its efficacy for non-alcoholic steatohepatitis (NASH) (Hall et al., 2019).

In 2021, Hahn MG et al. describe a new sGC activator runcaciguat (BAY1101042) that overcomes the limitations of first-generation sGC activators (Hahn et al., 2021). At present, runcaciguat has been found to have a kidney protective effect (Bénardeau et al., 2021; Stehle et al., 2022) and has certain efficacy in the treatment of non-proliferative diabetic retinopathy, which is in phase 2 clinical study (Sandner et al., 2021a). In addition, runcaciguat appears to have been found to enhance memory *in vivo* (Nelissen et al., 2022), which has the potential to be a new therapeutic direction for sGC activators.

Avenciguat is a novel sGC activator being developed for chronic kidney disease (CKD) and portal hypertension. It can reduce albuminuria in CKD patients and has good tolerability in chronic kidney disease (CKD) patients (Heerspink et al., 2024). Moreover, the current study results also support further studies of avenciguat in the treatment of portal hypertension (Lawitz et al., 2024).

MK-5475 is an inhaled sGC stimulator currently undergoing Phase I research. It can quickly reduce pulmonary vascular resistance in patients with PAH without inducing systemic vasodilation side effects, thus demonstrating potential lung selectivity (Bajwa et al., 2023). And subsequent research results also support further exploration of MK-5475 for the treatment of PH related to chronic obstructive pulmonary disease (COPD) (Bajwa et al., 2024). Another inhaled sGC activator, mosliciguat (BAY1237592), has also shown lung selection effects, which may overcome therapeutic limitations in patients with PH. And it is currently in phase I clinical development (Becker-Pelster et al., 2022).

Olinciguat is an sGC stimulator currently in phase 2 clinical development, which can exhibit pharmacological effects in various aspects (Zimmer et al., 2020). Currently, studies have shown that it can alleviate inflammation, vascular occlusion, and kidney damage in sickle cell disease (SCD) mice (Tchernychev et al., 2021).

Zagociguat (CY6463) is a novel central nervous system penetrative sGC stimulator primarily developed for neurodegenerative disorders. At present, it is still in the stage of I b research. Zagociguat has good clinical tolerability and can moderately lower blood pressure. Although no consistent pharmacological effects (PD) have been observed (van Kraaij et al., 2023a), current research results still support its further investigation (van Kraaij et al., 2023b).

BAY-747 is a long-acting sGC stimulator that has shown a hemodynamic effect up to 24 h in a Phase I study and can be used to treat refractory hypertension (Vakalopoulos et al., 2023; Wunder et al., 2023). There are also studies showing that BAY-747 appears to have a potential for Duchenne dystrophy (DMD) (Krishnan et al., 2021). In addition, BAY-747 also has the effect of enhancing memory in the body like runcaciguat (Nelissen et al., 2022).

In March 2024, Kintos, DP, et al. introduced A novel sGC stimulator (new multi-substituted 1H-pyrazolo [3,4-c] pyridin-7(6H)-ones) and highlighted that it is less likely to cause characteristic hypotension than other direct sGC stimulators (Kintos et al., 2024). If its clinical efficacy can be verified, it will cause a huge stir in the field and is likely to become the focus of future research.

### 4.5 Analysis of future development trends

Future research in this field is expected to continue its focus on the development of new drugs and the rigorous verification of their clinical efficacy and safety. It may be specifically reflected in the safety verification of riociguat, the effect of vericiguat on other types of heart failure, the clinical efficacy of praliciguat in diabetes nephropathy, and the efficacy and safety verification of several newly proposed compounds. Furthermore, actively exploring new therapeutic directions for sGC stimulators or activators may also emerge as a significant trend in the future development of this field.

### 4.6 Limitations

We applied bibliometric analysis to analyze the research trends on sGC stimulators or activators systematically and then visualized the findings in order to draw necessary conclusions. However, the study has some limitations: Firstly, we used WoSCC as the database for data collection. Although it is widely regarded as the most authoritative database, relying solely on one source may result in insufficient research. Secondly, when querying and downloading data from the WoSCC database, only general information can be accessed, but the full text of all papers cannot be downloaded, which may lead to a deviation in the analysis results. Thirdly, when querying the data, we have excluded papers published in 2024, as this may potentially lead to a slight lag in the results.

## 5 Conclusion

In this study, we included a total of 1,879 papers in our analysis. Since riociguat was approved for listing in 2013, the research on sGC stimulants or activators has always been a focus of attention for researchers, especially with the approval of vericiguat for market launch in 2021, which greatly increased researchers’ enthusiasm for research in this field.

Germany and the United States are the most foremost leaders and trailblazers in the development of this field. At the same time, Germany is also the exchange center of countries in the world, which greatly promotes the exchange and collision in the field of scientific research. BAYER AG is the institution that has made significant contributions to this field, participating in the research and development of various drugs, and driving the continuous updating and development of this field. PULMONARY CIRCULATION is the journal with the highest output, and the most cited journal is CIRCULATION. Professors Stasch, JP and Ghofrani, HA not only produce a greater volume of academic papers, but also wield significant influence in their field. They are widely recognized as authoritative scholars in this area. At present, the research focus in this field is mainly to study various drugs and explore their clinical efficacy and safety. The future research hotspots will focus on the following aspects based on the current research hotspots: the safety verification of riociguat, the clinical efficacy of vericiguat for other types of heart failure, the role of praliciguat in diabetes nephropathy, and the efficacy and safety of newly discovered drugs. Furthermore, actively exploring new therapeutic directions for sGC stimulators or activators may also be an important trend in the future development of this field.

## Data Availability

The original contributions presented in the study are included in the article/Supplementary Material, further inquiries can be directed to the corresponding authors.

## Footnotes

1 Total link strength: Total Link Strength refers to the sum of weights of all weighted links of a certain node in the network. Here, it represents the degree of closeness of the cooperative relationship between countries

## Abbreviations

sGC, soluble guanylate cyclase; WoSCC, Web of Science Core Collection; NO, nitric oxide; PH, pulmonary hypertension; HF, heart failure; HFrEF, heart failure with reduced ejection fraction; IF, Impact Factor; ISI, Institute for Scientific Information; SSc, systemic sclerosis; PA, pulmonary arterial hypertension; CTEPH, chronic thromboembolic pulmonary hypertension; HFpEF, heart failure with preserved ejection fraction; CTD-PAH, pulmonary hypertension associated with connective tissue disease; ADHF, acute decompensated heart failure; DN, diabetic nephropathy; DA, ductus arteriosus; NASH, non-alcoholic steatohepatitis; CKD, chronic kidney disease; COPD, chronic obstructive pulmonary disease; SCD, sickle cell disease; DMD, Duchenne dystrophy.
