# Supplementary material for: Analysis of research trends and development prospects of soluble guanylate cyclase stimulators/activators: using bibliometric methods
Source: Front Pharmacol. 2025 Jun 10;16:1501330. doi: 10.3389/fphar.2025.1501330 (PMC12185520; doi:10.3389/fphar.2025.1501330)
Supplement: Supplementary file 14 [file DataSheet1.docx]

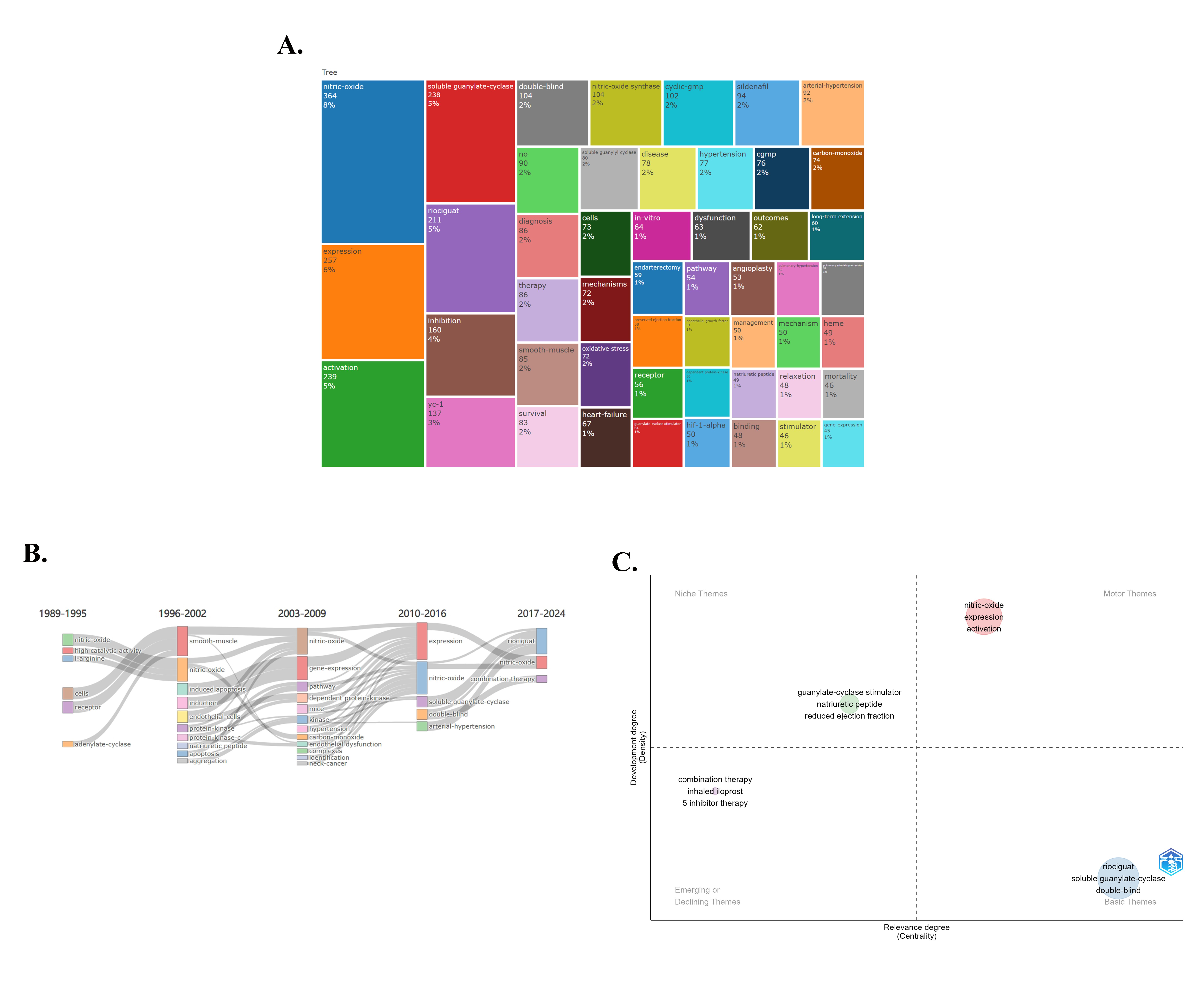


**Supplementary Figure 1** R software analysis of keywords. (**A.**) The keyword tree view of the top 50 keywords. (**B.**) The Sankey plot of keywords within different time slices. (**C.**) The keyword clustering of the fifth time slice (2017-2024).


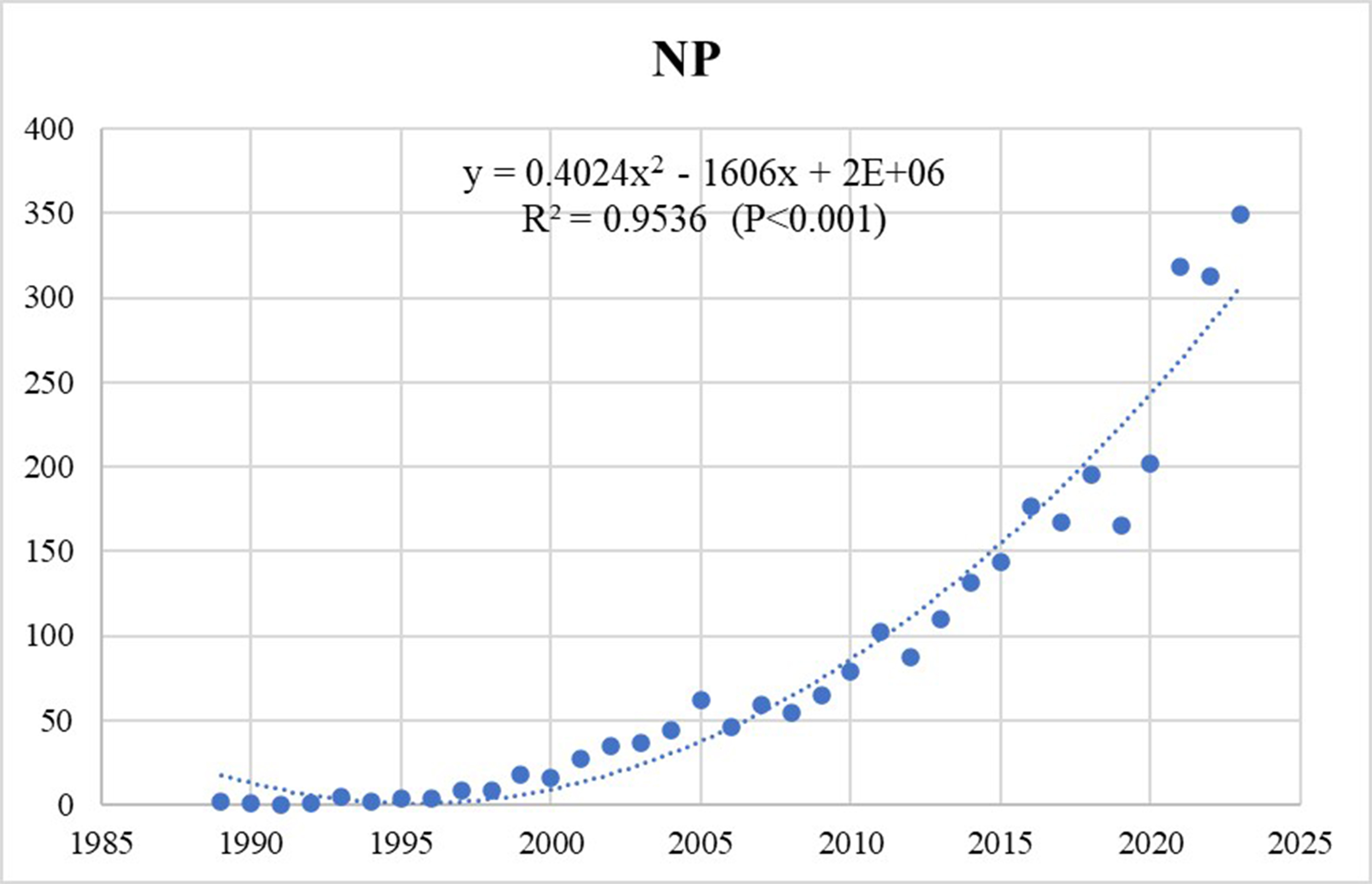


**Supplementary Figure 2** The scatter plot illustrating the annual publication volume (Based on the Scopus database)


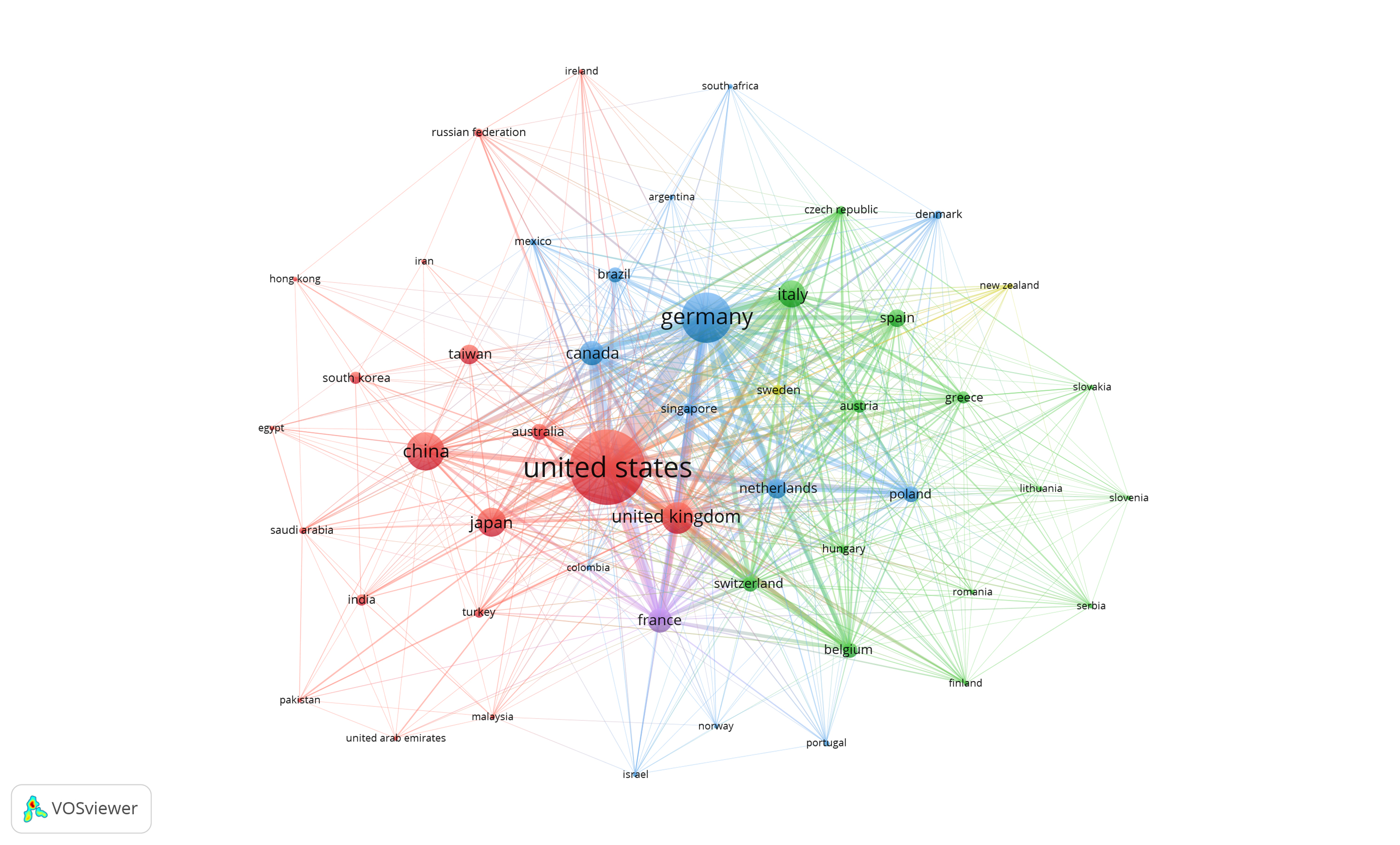


**Supplementary Figure 3** The visualization of the number of papers published by countries. Of the 190 countries,50 had published at least 5 papers. The size of the points in the figure represents the number of papers published by each country. The larger the point, the higher the number of papers published by the country indicated by it.(Based on the Scopus database)


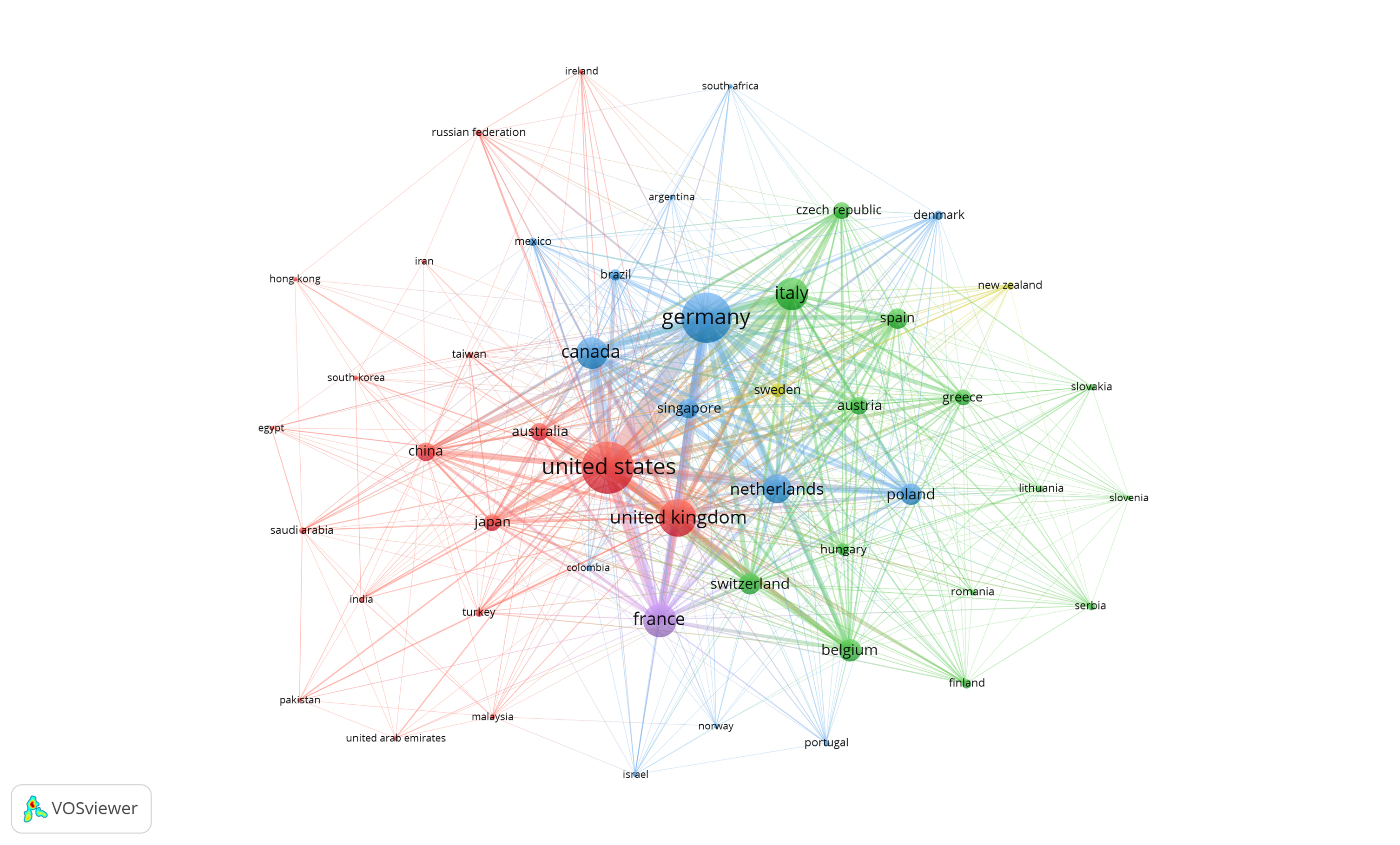


**Supplementary Figure 4** Network visualization of co-authorship countries. Of the 190 countries,50 had published at least 5 papers. The size of the dots in the figure represents the degree of closeness of each country's connection with other countries. The larger the dot, the closer the cooperation between this country and other countries. The lines connecting the dots represent a kind of cooperative relationship. The thicker the line, the closer the cooperation.(Based on the Scopus database)


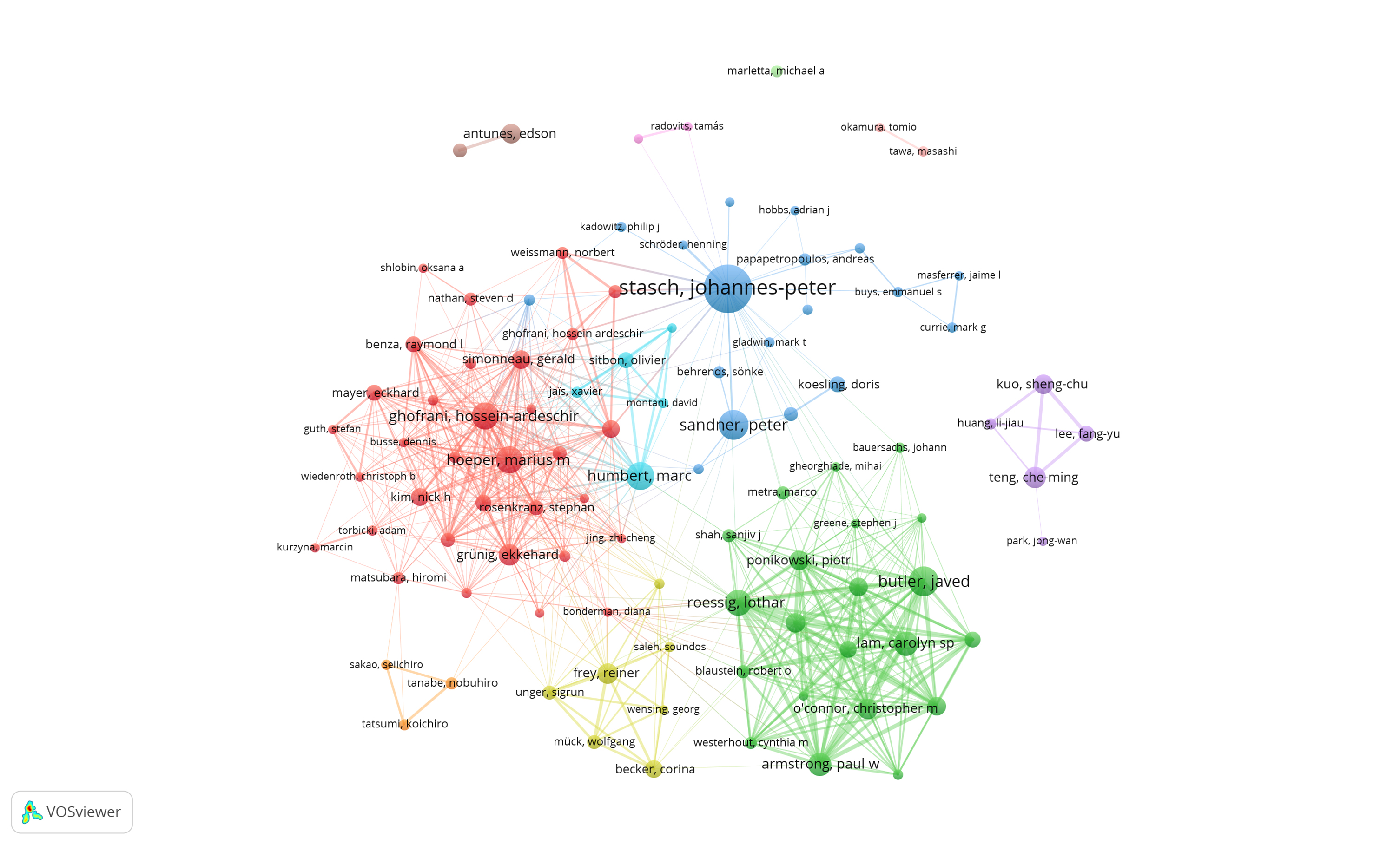


**Supplementary Figure 5** The visualization of the number of papers published by authors. Of the 12358 authors, 99 had published at least 10 papers. The size of the points in the figure represents the number of papers published by each author. The larger the point, the higher the number of papers published by the author indicated by it.(Based on the Scopus database)


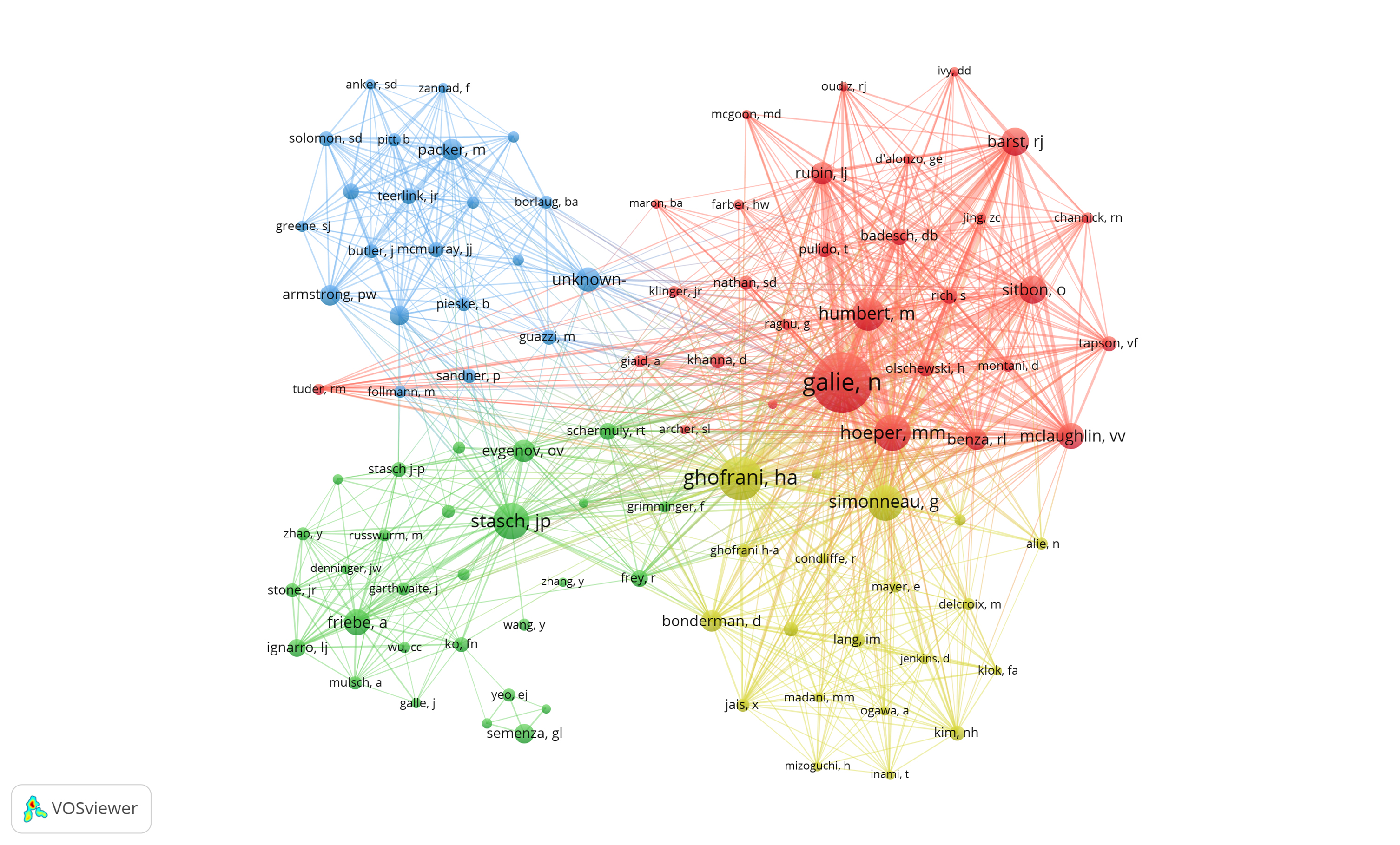


**Supplementary Figure 6** VOS viewer network visualization of co-cited authors. Of the 58447 co-cited authors,99 had at least 99 citations. The size of the points in the figure represents the frequency of citation of the authors, and the lines connecting the points indicate the co-citation relationship.(Based on the Scopus database)


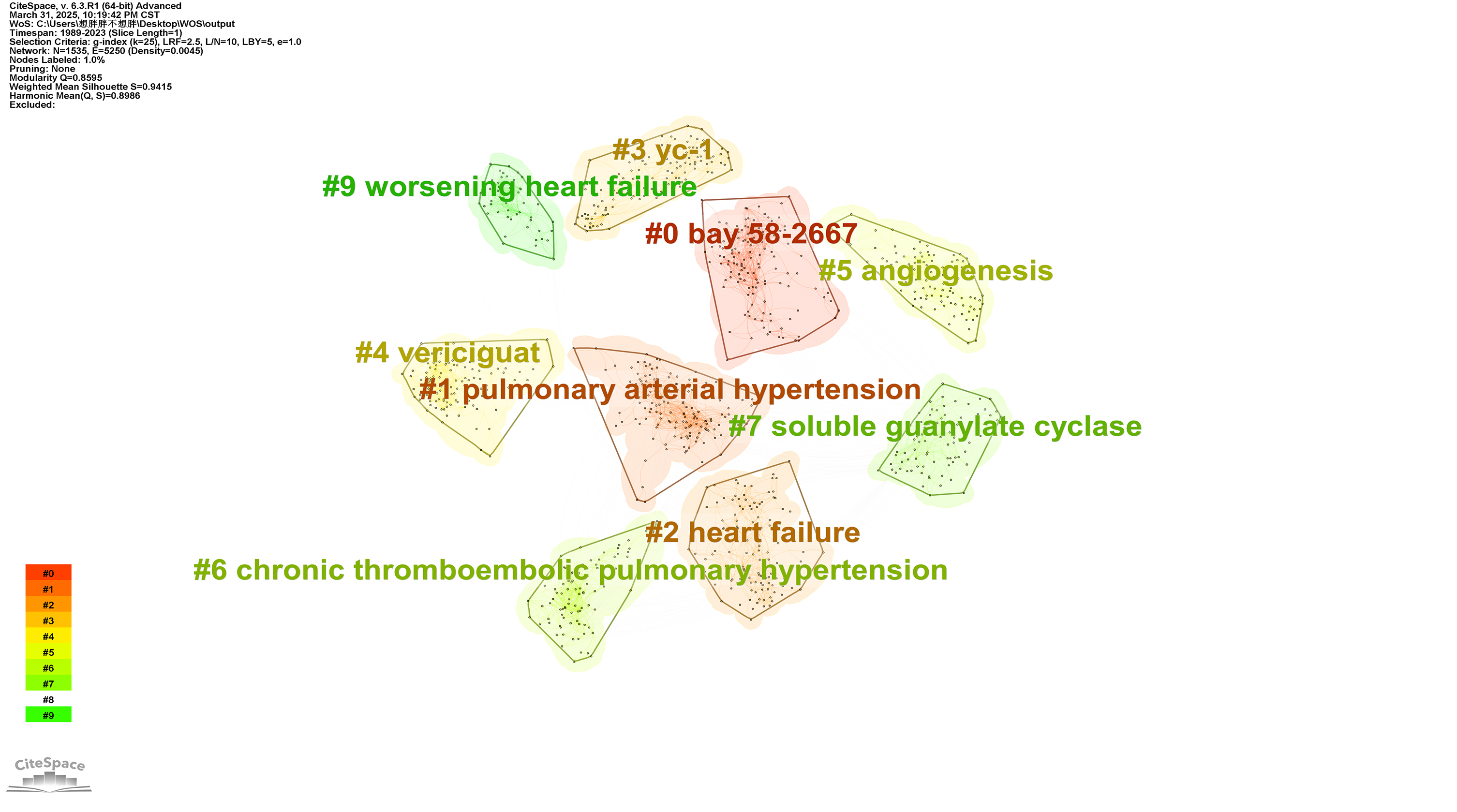


**Supplementary Figure 7** The cluster analysis of references. (Based on the Scopus database)


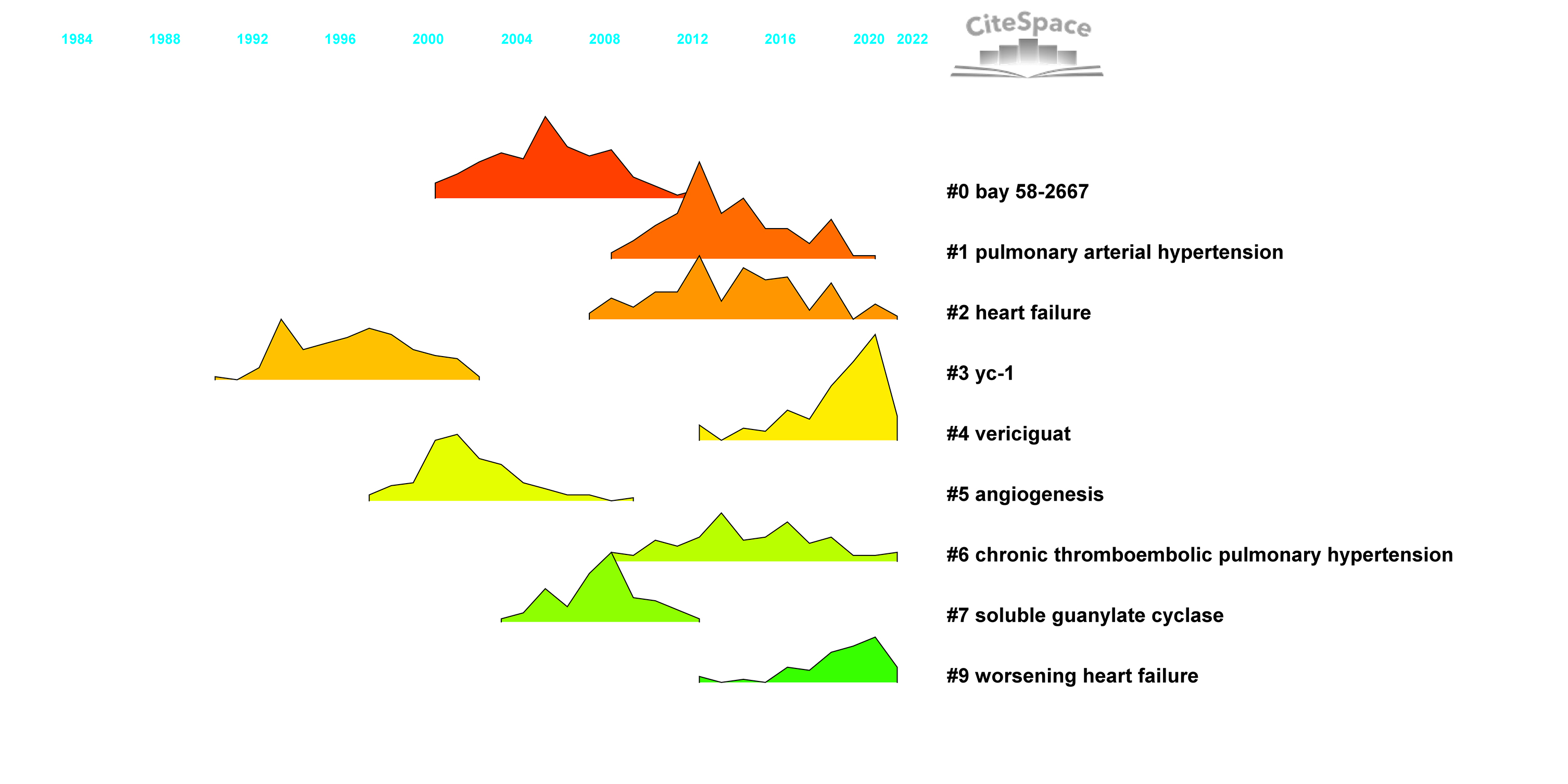


**Supplementary Figure 8** The landscape view of references. (Based on the Scopus database)


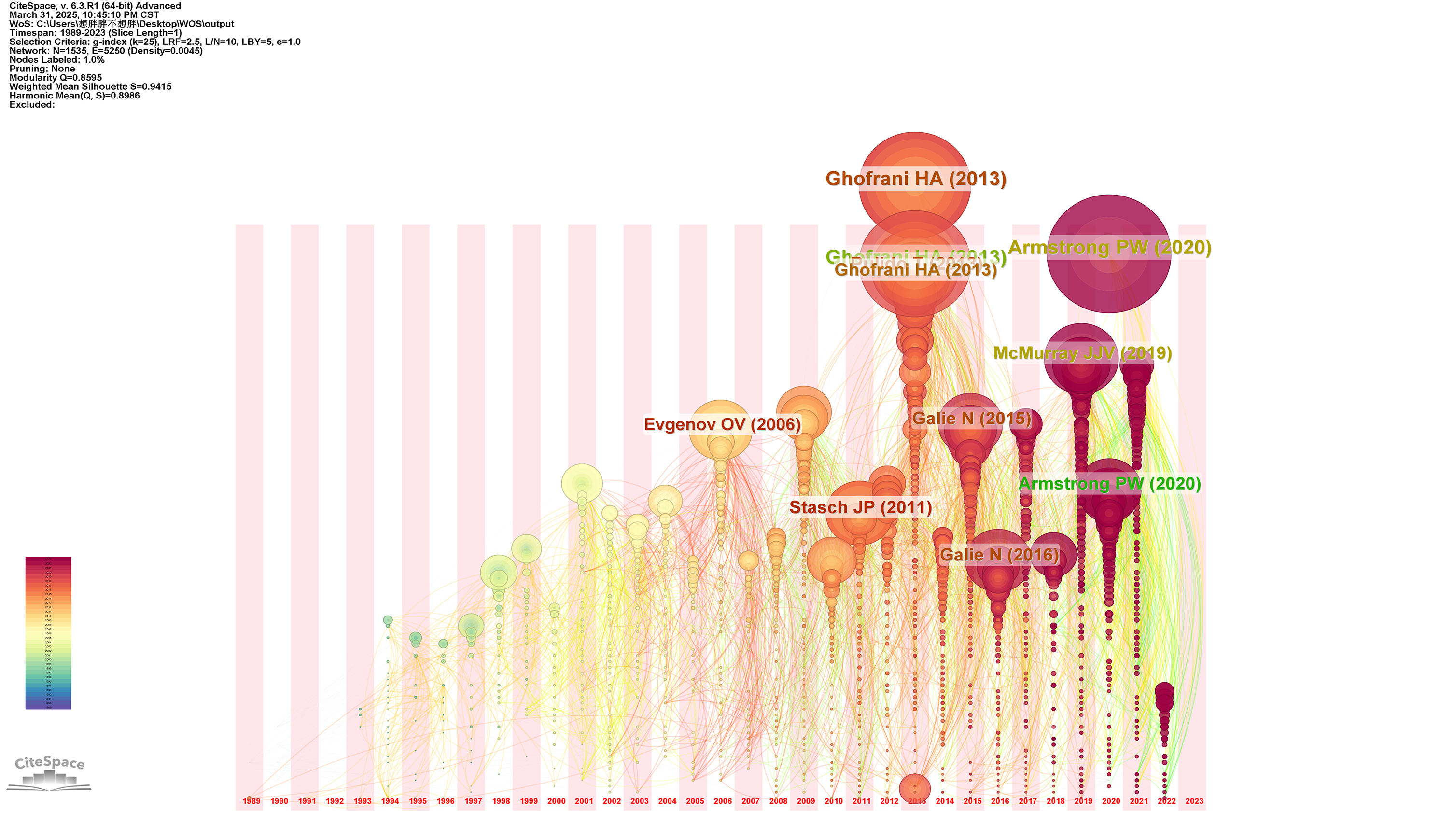


**Supplementary Figure 9** The time zone view of references. (Based on the Scopus database)


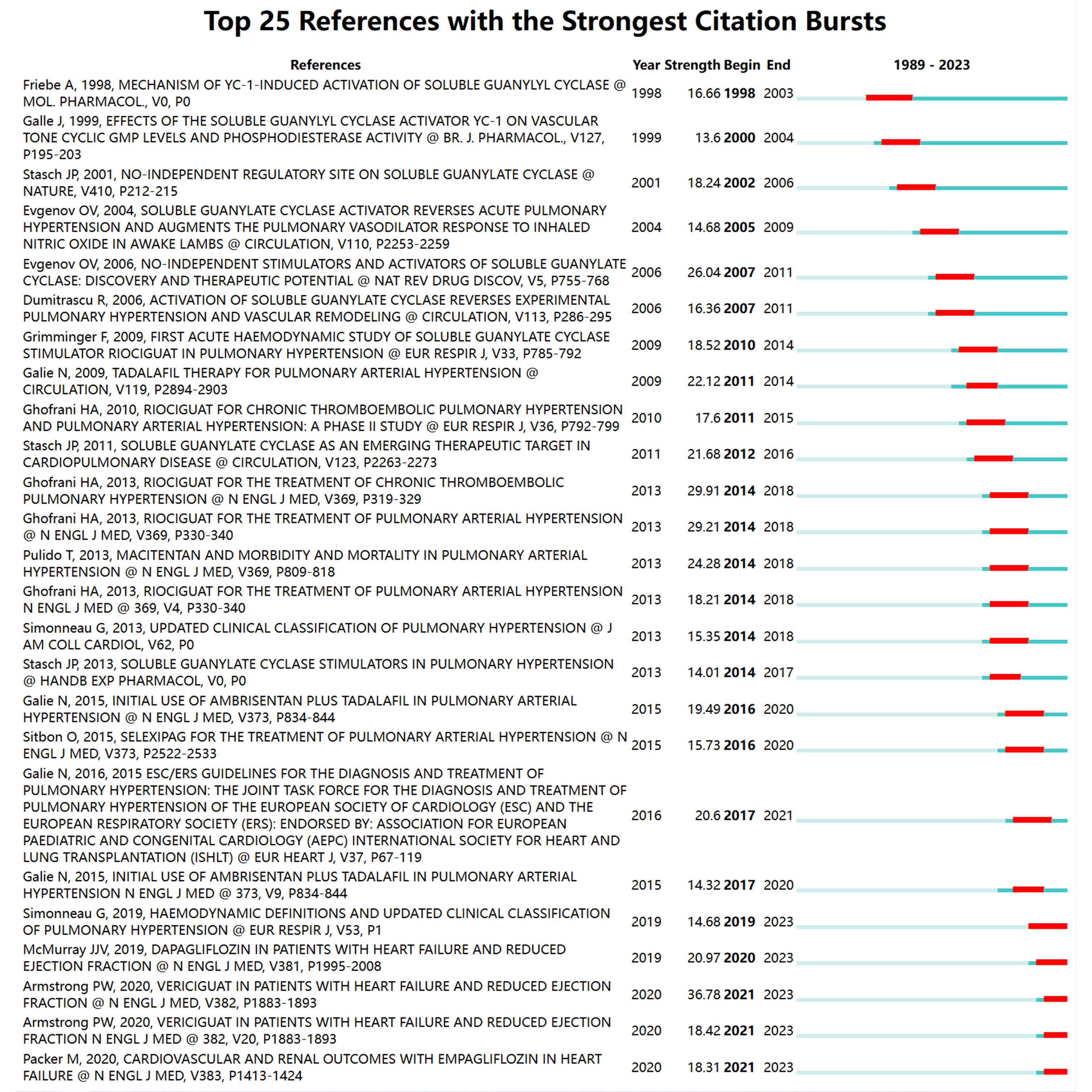


**Supplementary Figure 10** The top 25 references with the strongest citation bursts. (Based on the Scopus database)


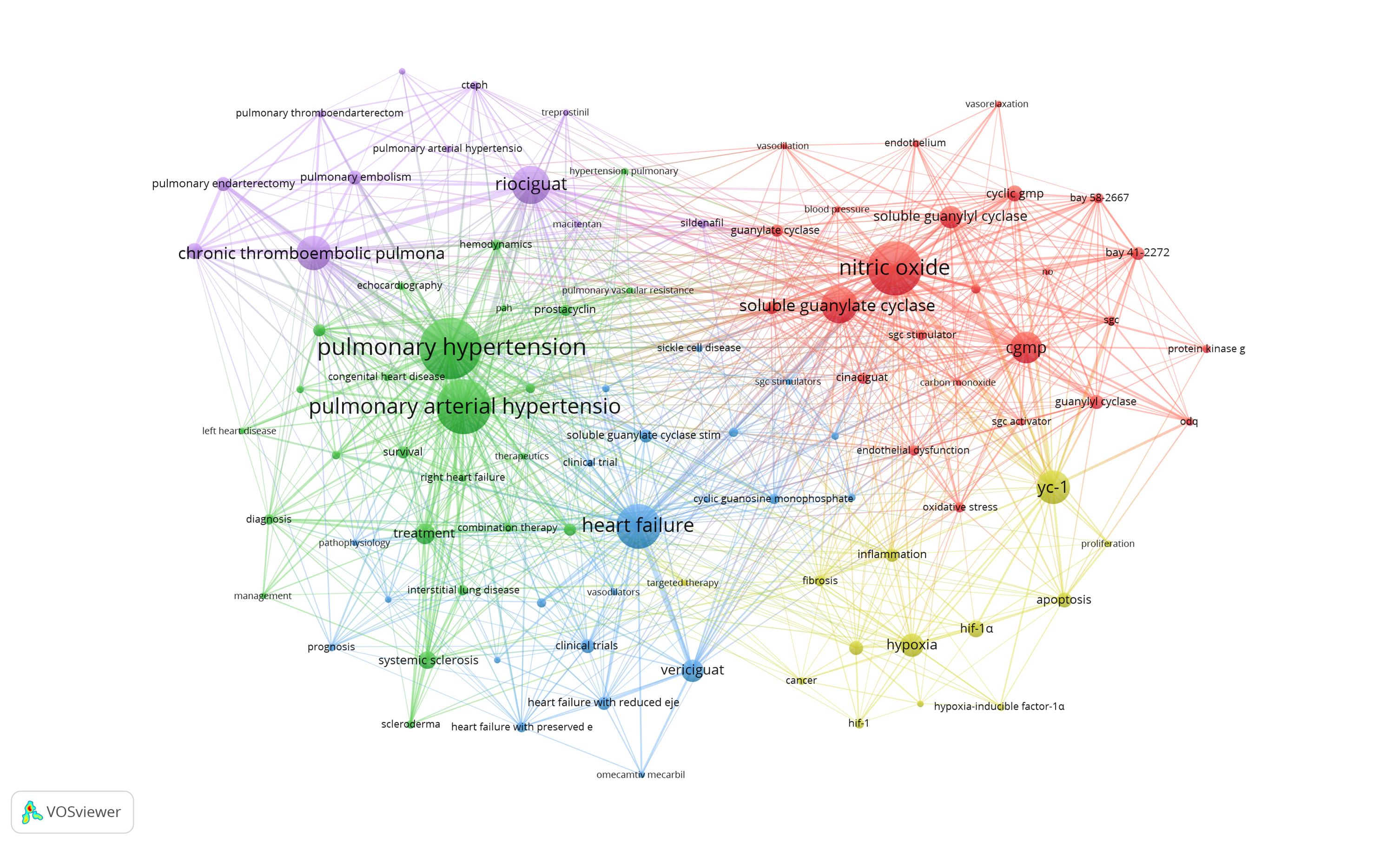


**Supplementary Figure 11** Network visualization of keywords. Of the 4857 keywords,100 had at least 14 occurrences. The size of each point in the figure represents the frequency of the occurrence of the corresponding keyword. The larger the point, the more times the keyword appears. Meanwhile, the lines connecting the points indicate the co-occurrence relationship, signifying that the two keywords appear simultaneously in the same article.(Based on the Scopus database)


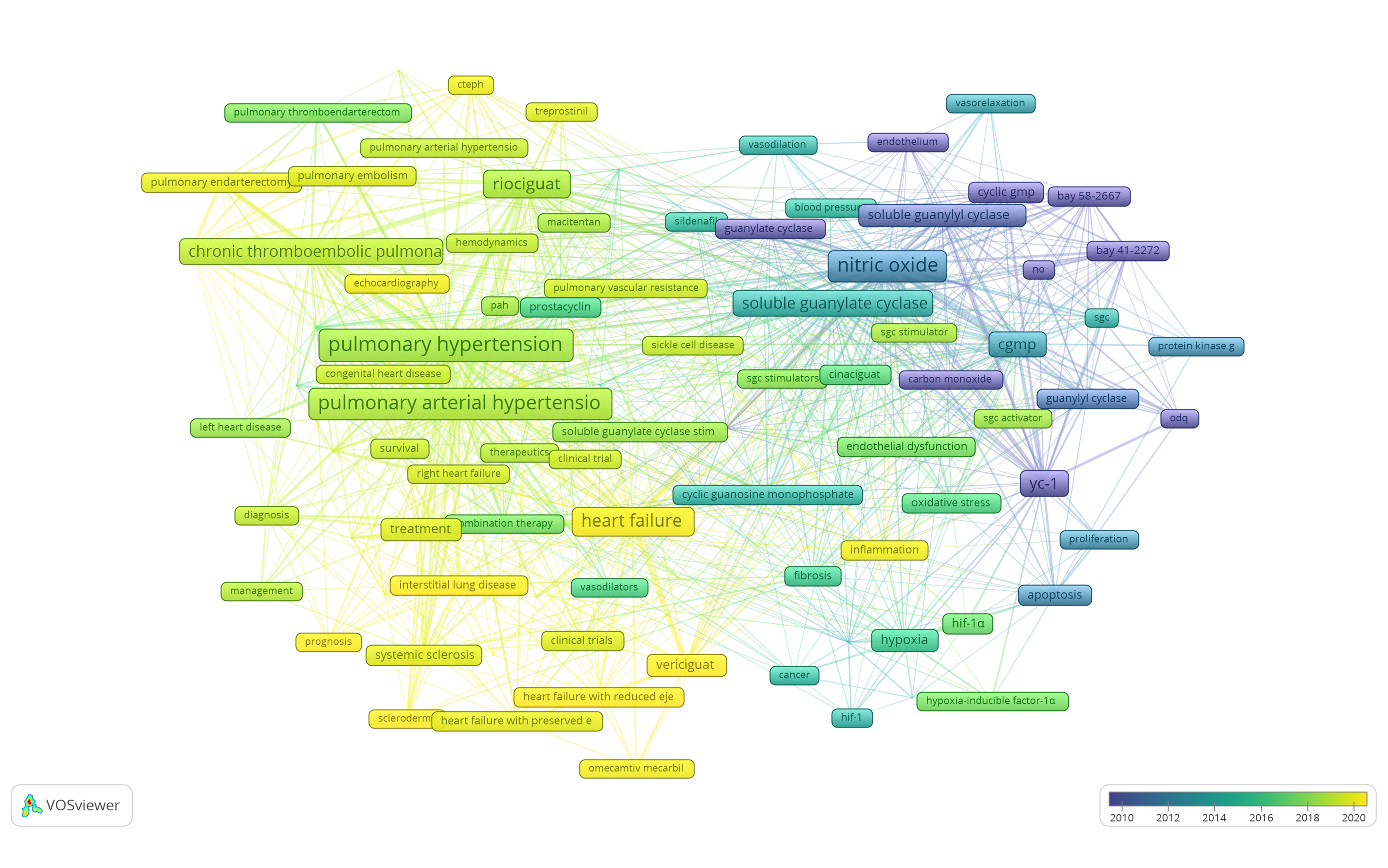


**Supplementary Figure 12** Overlay visualization of keywords. Of the 4857 keywords,100 had at least 14 occurrences. The color of the points in the figure indicates the time when the keywords appear. The warmer the color of the point is, the later the time when the keyword appears. (Based on the Scopus database)


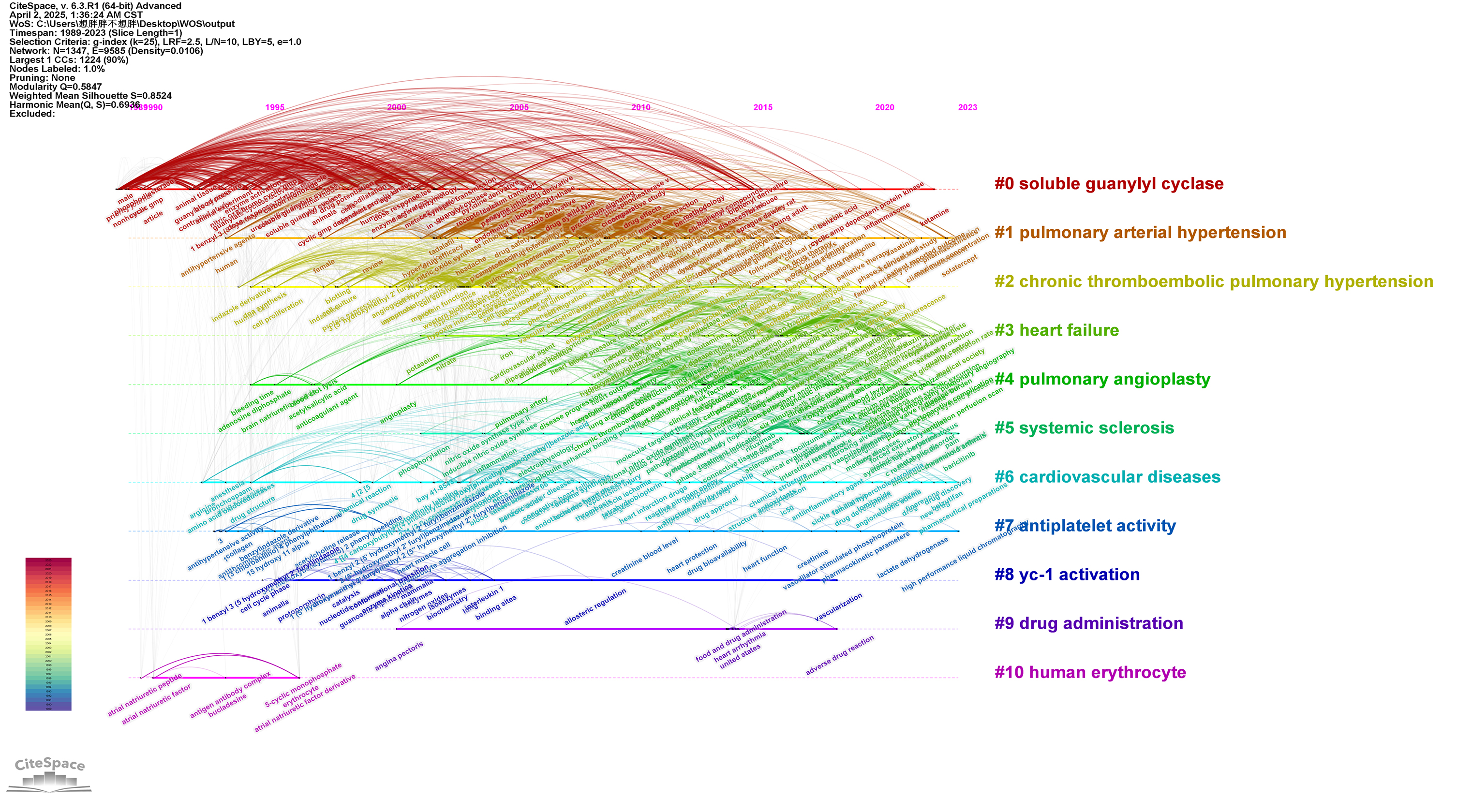


**Supplementary Figure 13** The timeline view of keywords. (Based on the Scopus database)


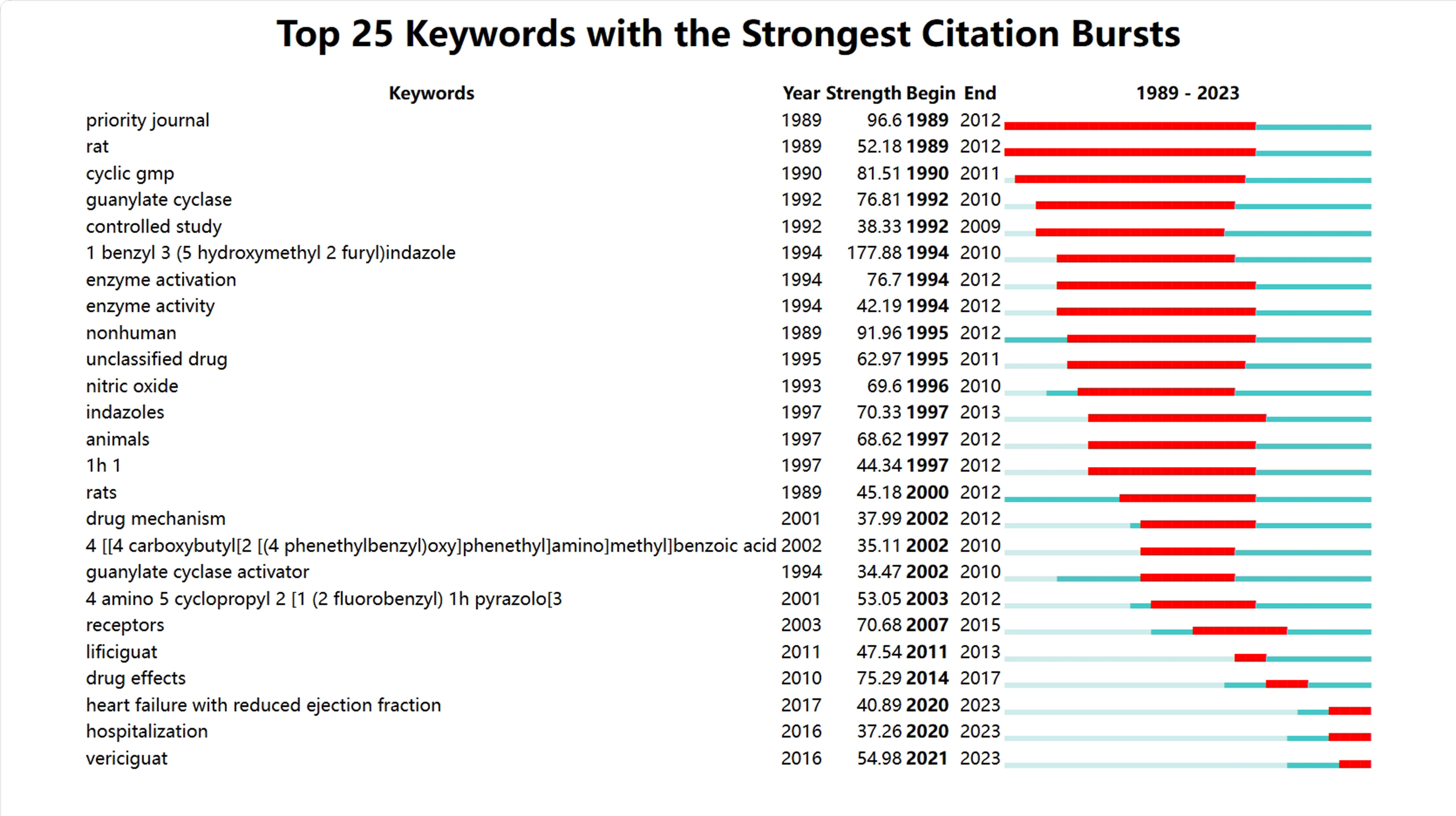


**Supplementary Figure 14** The top 25 keywords with the strongest citation bursts. (Based on the Scopus database)
